# Evaluating pediatric tuberculosis dosing guidelines: A model-based individual data pooled analysis

**DOI:** 10.1371/journal.pmed.1004303

**Published:** 2023-11-21

**Authors:** Lufina Tsirizani Galileya, Roeland E. Wasmann, Chishala Chabala, Helena Rabie, Janice Lee, Irene Njahira Mukui, Anneke Hesseling, Heather Zar, Rob Aarnoutse, Anna Turkova, Diana Gibb, Mark F. Cotton, Helen McIlleron, Paolo Denti

**Affiliations:** 1 Division of Clinical Pharmacology, Department of Medicine, University of Cape Town, Cape Town, South Africa; 2 Training and Research Unit of Excellence, Kamuzu University of Health Sciences, Blantyre, Malawi; 3 Department of Pediatrics, University of Zambia, School of Medicine, Lusaka, Zambia; 4 University Teaching Hospitals-Children’s Hospital, Lusaka, Zambia; 5 Department of Pediatrics and Child Health and Family Center for Research with Ubuntu, Stellenbosch University, Cape Town, South Africa; 6 Drugs for Neglected Diseases initiative, Geneva, Switzerland; 7 Desmond Tutu TB Centre, Department of Pediatrics and Child Health, Faculty of Medicine and Health Sciences, Stellenbosch University, Cape Town, South Africa; 8 Department of Pediatrics and Child Health, Red Cross War Memorial Children’s Hospital, and SA-MRC Unit on Child & Adolescent Health, University of Cape Town, Cape Town, South Africa; 9 Radboud University Medical Center, Nijmegen, the Netherlands; 10 Medical Research Council Clinical Trials Unit at University College London, Institute of Clinical Trials and Methodology, London, United Kingdom; 11 Wellcome Centre for Infectious Diseases Research in Africa (CIDRI-Africa), Institute of Infectious Disease and Molecular Medicine, University of Cape Town, Cape Town, South Africa; University of Southampton, UNITED KINGDOM

## Abstract

**Background:**

The current World Health Organization (WHO) pediatric tuberculosis dosing guidelines lead to suboptimal drug exposures. Identifying factors altering the exposure of these drugs in children is essential for dose optimization. Pediatric pharmacokinetic studies are usually small, leading to high variability and uncertainty in pharmacokinetic results between studies. We pooled data from large pharmacokinetic studies to identify key covariates influencing drug exposure to optimize tuberculosis dosing in children.

**Methods and findings:**

We used nonlinear mixed-effects modeling to characterize the pharmacokinetics of rifampicin, isoniazid, and pyrazinamide, and investigated the association of human immunodeficiency virus (HIV), antiretroviral therapy (ART), drug formulation, age, and body size with their pharmacokinetics.

Data from 387 children from South Africa, Zambia, Malawi, and India were available for analysis; 47% were female and 39% living with HIV (95% on ART). Median (range) age was 2.2 (0.2 to 15.0) years and weight 10.9 (3.2 to 59.3) kg. Body size (allometry) was used to scale clearance and volume of distribution of all 3 drugs. Age affected the bioavailability of rifampicin and isoniazid; at birth, children had 48.9% (95% confidence interval (CI) [36.0%, 61.8%]; *p* < 0.001) and 64.5% (95% CI [52.1%, 78.9%]; *p* < 0.001) of adult rifampicin and isoniazid bioavailability, respectively, and reached full adult bioavailability after 2 years of age for both drugs. Age also affected the clearance of all drugs (maturation), children reached 50% adult drug clearing capacity at around 3 months after birth and neared full maturation around 3 years of age. While HIV per se did not affect the pharmacokinetics of first-line tuberculosis drugs, rifampicin clearance was 22% lower (95% CI [13%, 28%]; *p* < 0.001) and pyrazinamide clearance was 49% higher (95% CI [39%, 57%]; *p* < 0.001) in children on lopinavir/ritonavir; isoniazid bioavailability was reduced by 39% (95% CI [32%, 45%]; *p* < 0.001) when simultaneously coadministered with lopinavir/ritonavir and was 37% lower (95% CI [22%, 52%]; *p* < 0.001) in children on efavirenz. Simulations of 2010 WHO-recommended pediatric tuberculosis doses revealed that, compared to adult values, rifampicin exposures are lower in most children, except those younger than 3 months, who experience relatively higher exposure for all drugs, due to immature clearance. Increasing the rifampicin doses in children older than 3 months by 75 mg for children weighing <25 kg and 150 mg for children weighing >25 kg could improve rifampicin exposures. Our analysis was limited by the differences in availability of covariates among the pooled studies.

**Conclusions:**

Children older than 3 months have lower rifampicin exposures than adults and increasing their dose by 75 or 150 mg could improve therapy. Altered exposures in children with HIV is most likely caused by concomitant ART and not HIV per se. The importance of the drug–drug interactions with lopinavir/ritonavir and efavirenz should be evaluated further and considered in future dosing guidance.

**Trial registration:**

ClinicalTrials.gov registration numbers; NCT02348177, NCT01637558, ISRCTN63579542

## Abstract 2

Lufina Galileya and colleagues pooled data from large pharmacokinetic studies to identify key covariates influencing drug exposure to optimize tuberculosis dosing in children.

## Author summary

**Why was this study done?:**

- Recent pharmacokinetic studies in children on 2010 World Health Organization (WHO)-recommended first-line tuberculosis treatment found variable exposures of rifampicin, isoniazid, and pyrazinamide, with rifampicin being the drug achieving the lowest exposures.
- Findings of the association of human immunodeficiency virus (HIV) with the exposures of first-line tuberculosis drugs are inconsistent, and the relationships of HIV, antiretrovirals, formulation, demographics, and different sample processing and analysis methods are unclear.
- To rapidly mitigate the low rifampicin exposures, policymakers have been looking to investigate if taking the existing fixed dose combination tablet along with an additional 75 mg or 150 mg tablet of rifampicin alone (top-up) would be a good way to optimize rifampicin doses in children.

**What did the researchers do and find?:**

- We pooled individual pharmacokinetic data from 3 large studies, which gave us a total of 387 children through whom we investigated the association of HIV, antiretrovirals, and formulation, and investigated the exposures achieved after increasing rifampicin doses by 75 mg or 150 mg.
- Age affected drug bioavailability and clearance and there was an association between body size and both the volume of distribution and drug clearance of first-line tuberculosis drugs.
- HIV had no association with the exposures of first-line tuberculosis drugs; rather, the antiretroviral lopinavir/ritonavir increased rifampicin exposure and reduced isoniazid and pyrazinamide exposure.
- Increasing rifampicin doses can lead to rifampicin exposures comparable to those observed in adults, and we report large differences between rifampicin and isoniazid formulations with reduced exposures in some formulations.

**What do these findings mean?:**

- There are opportunities to rapidly improve first-line pediatric tuberculosis treatment through increasing rifampicin doses, which can be done through a rifampicin tablet top-up to the current fixed dose combination tablet as part of the WHO guidelines.
- Future studies investigating the association of HIV with tuberculosis drug exposures should explore the relationship of different antiretroviral therapies with tuberculosis-drug exposures, including drug–drug interactions due to administration of the drugs at the same time.
- Where possible, children on HIV and tuberculosis co-treatment should be closely monitored for possible toxicities or reduced efficacy arising from drug–drug interactions.
- The main limitation of our study is the small study design differences among the pooled studies, which led to some unattributable associations that we classified as site or study associations.

## Introduction

Children account for 11% of global tuberculosis cases [1]. Approximately 1.1 million children develop tuberculosis disease and 210,000 die of tuberculosis complications each year [1,2]. Human immunodeficiency virus (HIV) increases the risk for active tuberculosis disease 20-fold. HIV and tuberculosis coinfection and co-medication are, therefore, common [3].

Diagnosing and treating tuberculosis in children is challenging because of the limited diagnostics and restricted treatment options in this population. The World Health Organization (WHO) recommends first-line tuberculosis treatment for children, comprising rifampicin 15 (10 to 20) mg/kg, isoniazid 10 (7 to 15) mg/kg, and pyrazinamide 35 (30 to 40) mg/kg with or without ethambutol 20 (15 to 25) mg/kg. This regimen is given to children with drug-susceptible tuberculosis for 2 months, followed by 4 months of rifampicin and isoniazid [4]. Because of potential drug–drug and drug–disease interactions, children with HIV and tuberculosis coinfection are at risk of suboptimal exposures of tuberculosis drugs [5–7].

Previous studies have reported contradictory findings on the association of HIV on the pharmacokinetics of tuberculosis drugs in both children and adults [5]. Some antiretroviral therapies (ART) have been reported to alter the exposure of tuberculosis drugs in adults. For example, efavirenz has been reported to increase isoniazid clearance by 24%, thereby reducing its exposure [6]. Ritonavir was reported to decrease rifampicin clearance by 46%, increasing its exposure [7]. There is a paucity of data to support dose adjustments to negate the associations of HIV and ART on the exposure of first-line tuberculosis drugs in children. The existing studies are small, probably due to the ethical regulations surrounding recruiting children in pharmacokinetic clinical trials and the high cost of conducting them [8].

Reduced antituberculosis drug exposures, which may result from, for example, faulty formulations, drug–drug interactions, or not accounting for age and body size, may reduce efficacy, leading to treatment failure or relapse, with an increased risk of drug resistance requiring longer, complex, and more expensive treatment [9]. On the other hand, overexposure increases the risk of toxicity [10]. It is therefore essential that childhood tuberculosis dosing is optimized for all children, including those coinfected with HIV.

Understanding and quantifying the associations of HIV, ART, drug formulation, age, and body size on the pharmacokinetics of tuberculosis drugs is essential to optimizing treatment in children with tuberculosis and HIV coinfection. However, conducting clinical trials to answer these questions is challenging and expensive [8]. Pooling datasets of existing pediatric clinical studies is therefore a good approach to answering these questions and can increase the power to find relationships that smaller studies cannot. We conducted a pooled pharmacokinetic analysis of individual pediatric patient data from 3 multisite studies conducted in South Africa, Malawi, India, and Zambia. We aimed to characterize the pharmacokinetics of first-line tuberculosis drugs, quantify the association of HIV, ART, age, body size, and drug formulation on the exposures of these drugs, and evaluate the current WHO-recommended pediatric tuberculosis dosing guidelines in children.

## Methods

### Study design and setting

The first study included the pooled analysis was SHINE (Clinical trial registration number ISRCTN63579542), a randomized controlled trial of therapy shortening for minimal tuberculosis with 2010 WHO-recommended doses using fixed-dose combination (FDC) drugs in African and Indian children with or without HIV. Children were randomized to either 4 or 6 months (2 months intensive phase in each case) first-line tuberculosis treatment [11].

The second study, conducted by Drugs for Neglected Diseases initiative (DNDi) (Clinical trial registration number NCT02348177), was a Phase I/II open-label, sequential non-randomized study comparing pharmacokinetics of lopinavir boosted with ritonavir (4:1 ratio) without rifampicin versus lopinavir super-boosted with ritonavir (4:4 ratio) in the presence of rifampicin in children [12].

DATiC (Clinical trial registration number NCT01637558) was a pharmacokinetic study investigating optimal dosing of first-line antituberculosis and ART drugs in children. On the day of pharmacokinetic sampling, single (not co-formulated) tuberculosis-drug formulations were used in doses according to WHO 2010 guidelines. Before and after pharmacokinetic evaluation, the standard of care treatment was delivered using older WHO guidelines for FDCs available in the public health sector at the time [13]. In all 3 studies, study drugs were administered by the study staff on the day of pharmacokinetic sampling. More details about the studies included in this analysis are in Table 1.

**Table 1:** Characteristics of studies included in the pooled analysis.

Study	Study period	Sites	Age*	Weight*	HIV status*	Health condition*	TB-drug formulations	Pharmacokinetic sampling day(s)	Pharmacokinetic sampling schedule
**SHINE** [11]	July 2016 to July 2018	i) Cape Town, South Africa ii) Lusaka, Zambia iii) Chennai & Pune in India	0–16 years	≥3 kg	HIV+ and HIV-	Children with minimal TB**	Pediatric and adult FDC from Macleod’s Pharmaceuticals Ltd	i) HIV-: 2–8 weeks after starting treatment ii) HIV+: during the last month of continuation phase of TB treatment	Pre-dose, 1, 2, 4, 6, 8, and 12 hours post-dose
**DNDi** [12]	January 2013 to November 2015	i) Johannesburg ii) Cape Town iii) Durban South Africa	>42 weeks gestational age	3–15 kg	HIV+	Children with TB	Pediatric FDC of rifampicin and isoniazid from Sandoz Pharmaceuticals Ltd Single pediatric pyrazinamide tablet from Sandoz Pharmaceuticals Ltd	During second month of co-treatment with rifampicin, during the last month of co-treatment and 4–6 weeks after rifampicin treatment	Pre-dose, 1, 2, 4, 6, and 10 hours post-dose
**DATiC** [13]	November 2012 to June 2017	i) Blantyre, Malawi ii) Cape Town, South Africa	<12 years	1.5–30 kg	HIV+ and HIV-	Children with TB (those with acute severe illness excluded)	On day of pharmacokinetic sampling; Rifampicin: i) Eremfat, Riemser Arzneimittel, Germany ii) R-Cin Aspen Pharmacare, South Africa iii) Rimactazid, Novartis, India. Isoniazid: i) Riemser Arzneimittel, Germany ii) Rimactazid, Novartis, India. Pyrazinamide: i) Svizera Laboratories, India	At least 2 weeks after starting intensive TB treatment phase	Pre-dose, 1, 2, 4, 6, 8, and 10 hours post-dose

*Study inclusion criteria.

**Defined as respiratory tuberculosis confined to 1 lobe (opacification of <1 lobe) with no cavities, no signs of miliary tuberculosis, no complex pleural effusion, and no clinically significant airway obstruction or peripheral lymph-node tuberculosis.

DNDi, Drugs for Neglected Diseases initiative; FDC, fixed-dose combination; HIV, human immunodeficiency virus; TB, tuberculosis.

### Ethics statement

We pooled individual patient data from 3 multisite studies of children on the 2010 WHO-recommended first-line tuberculosis-drugs, summarized in Table 1. The DATiC study protocol was approved by the Health Research Ethics Committees of the Universities of Stellenbosch and Cape Town and the College of Medicine Research Ethics Committee in Malawi. The DNDi study protocol was approved by the ethics committees of Stellenbosch University, University of Cape Town, University of the Witwatersrand, and Pharma Ethics in Durban. The SHINE trial was approved by the Ethics Committees of the all participating sites in South Africa (Stellenbosch University and University of Cape Town Research Ethics Committees), Uganda (Joint Clinical Research Centre Institutional Review Board), Zambia (University of Zambia Biomedical Research Ethics Committee and National Health Research Ethics Committee), India (BJMC Ethics Committee-Pune, National Institute of Research in Tuberculosis Ethics Committee-Chennai and Health Ministry Screening Committee), and by University College London Research Ethics Committee. For all 3 studies, parents or legal guardians provided written informed consent and older children provided written assent.

### Study sample analyses

Rifampicin, isoniazid, and pyrazinamide plasma concentrations were quantified using validated liquid chromatography with tandem mass spectrometry (LC-MS/MS) methods at University of Cape Town (UCT) Division of Clinical Pharmacology, with the exception of SHINE samples from India (5% of all samples), which were assayed using validated LC-MS/MS at National Institute of Research in Tuberculosis in India [14]. Three India-based participants’ drug concentrations (21 samples) were assayed at both UCT and India for comparison. We adjusted for the overall difference in analysis methods of the 2 laboratories during pharmacokinetic model development. Assays at the UCT laboratory had a lower limit of quantification (LLOQ) of 0.117 mg/L for rifampicin, 0.0977 mg/L for isoniazid, and 0.200 mg/L for pyrazinamide. The LLOQ was 0.250 mg/L for all 3 drugs at the laboratory in India.

### Population pharmacokinetic analysis

We analyzed concentration-time data using nonlinear mixed-effects modeling with NONMEM version 7.4.4 (ICON Development Solutions, Ellicott City, Maryland, United States of America). We applied first-order conditional estimation with interaction (FOCE-I) and stochastic approximation expectation-maximization (SAEM) to estimate population pharmacokinetic parameters. To visualize data, we utilized Perl-speaks-NONMEM (PsN version 4.7.0), Pirana version 2.9.9, and R version 3.6.3 [15].

Samples below LLOQ (BLQ) were handled as described previously [13,16]. In short, 50% of the LLOQ value was imputed for censored values. For the first censored value closest to the peak concentration, the additive error was inflated by LLOQ/2, and all trailing censored values were excluded from the model fit but retained for simulation-based diagnostics. In the SHINE (samples from India) and DATiC studies, values between the LLOQ and lower limit of detection (LOD) were available, and these were used as such. Data below LOD were handled as described above.

We tested one- and two-compartment disposition models with first-order absorption with either transit compartments or lag time. We explored first-order elimination for the 3 drugs as well as first-pass and saturable elimination for rifampicin.

We included between-subject variability (BSV) on disposition parameters and between-occasion variability (BOV) on absorption parameters assuming a log-normal distribution. We described residual unexplained variability with a combined additive and proportional error model. To account for body size differences, we allometrically scaled (with either weight or fat-free mass) clearances and volumes of distribution with exponents of 0.75 and 1, respectively [17]. We investigated the association of age with clearance and bioavailability for all drugs and employed prior information to estimate the maturation of isoniazid and rifampicin clearance [18]. After including weight and age in the model, we tested additional covariates based on physiological plausibility, range of covariate values in the dataset, and assessment of empirical Bayes estimates versus covariate plots. N-acetyltransferase 2 (NAT2) acetylator-status (important for isoniazid clearance [19]) was available for 142 participants in the DATiC study. We applied a mixture-model for participants with unknown acetylator-status [20], fixed the proportions of fast, intermediate, and slow metabolizers to values reported previously in a study with a bigger sample size, and estimated the respective clearances [19]. We maintained covariates reaching statistical significance of *p* < 0.05 on forward addition and *p* < 0.01 on backward elimination. *P*-values were derived using the likelihood ratio test.

We modeled the datasets sequentially, attempting to correct for any systematic differences between studies, as previously suggested [21]. We assessed performance of intermediate and final models with visual predictive checks (VPCs) and evaluated parameter precision using a sampling importance resampling (SIR) procedure.

### Simulations

**Fig 1:**
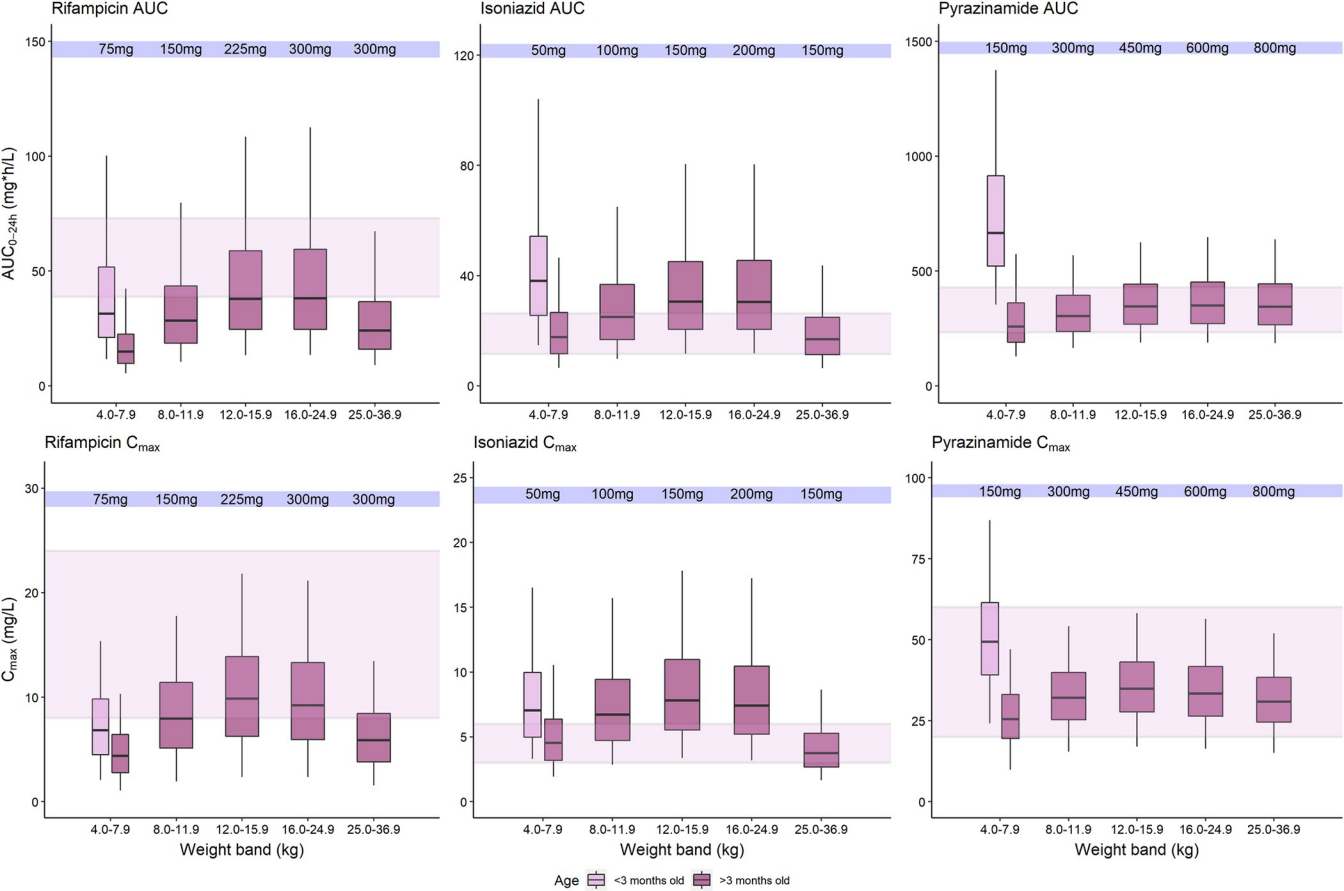
Simulated drug exposures after administration of WHO recommended doses and weight bands. Simulated rifampicin, isoniazid, and pyrazinamide area under the curve from time zero to 24 h (AUC_0-24_) after dose and maximum concentrations (C_max_) at steady state, versus body weight, with concentrations achieved after administration of the WHO-recommended doses and weight-bands with the existing FDC formulation. The maroon boxes represent patients <3 months old while the pink boxes represent patients ≥3 months. The shaded area represents the range of the reported adult median C_max_ and AUC_0-24._ The box indicates the interquartile range, while the whiskers denote the 2.5th and the 97.5th percentiles. Figure A in S1 Text shows WHO-recommended doses and weight-bands with weight stratified per kilogram. FDC, fixed-dose combination; WHO, World Health Organization.

**Fig 2:**
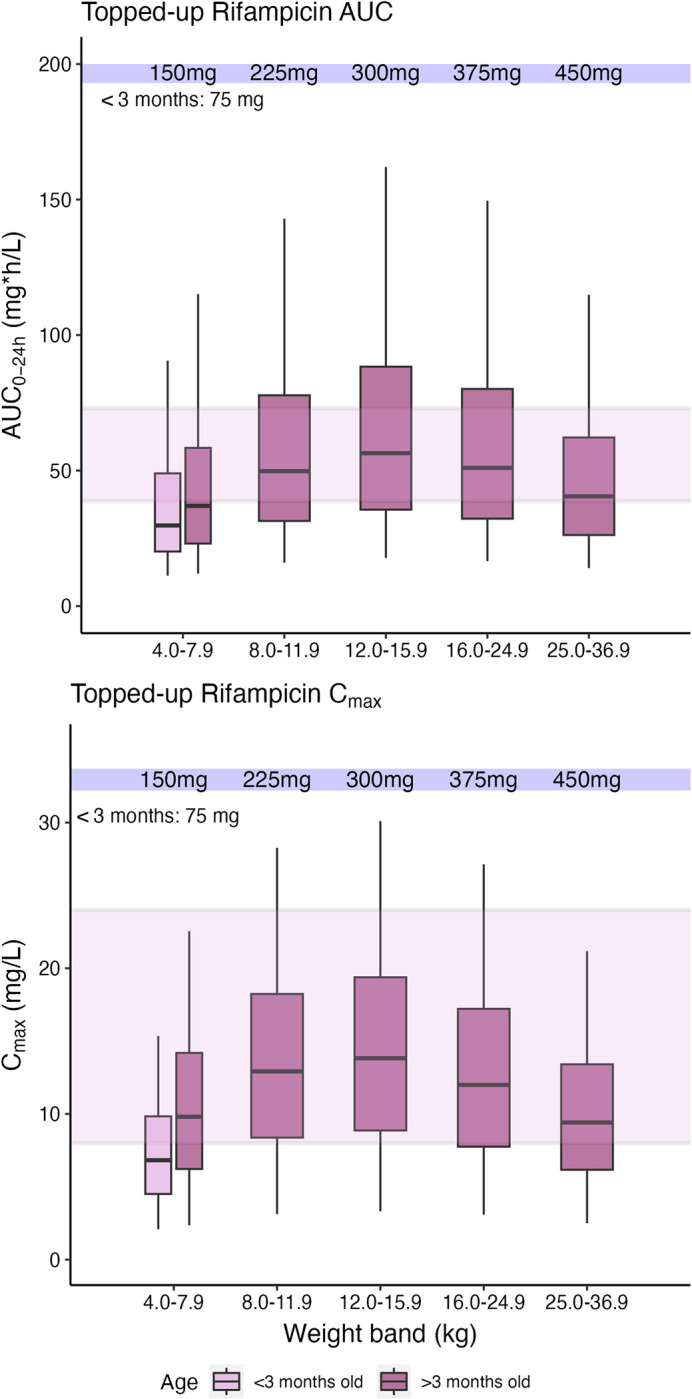
Simulated rifampicin exposures after adding 75 mg or 150 mg to the WHO recommended doses and weight bands. Simulated rifampicin area under the curve from time zero to 24 h (AUC_0-24_) after dose and maximum concentrations (C_max_) at steady state, versus body weight, with concentrations achieved after topping up the existing FDC formulation with 75 mg and 150 mg tablets of rifampicin alone in children weighing <25 kg and >25 kg, respectively, using the WHO-recommended doses and weight-bands. The maroon boxes represent patients <3 months old while the pink boxes represent patients ≥3 months. The shaded area represents the range of the reported adult median C_max_ and AUC_0-24._ The box indicates the interquartile range, while the whiskers denote the 2.5th and the 97.5th percentiles. FDC, fixed-dose combination; WHO, World Health Organization.

**Fig 3:**
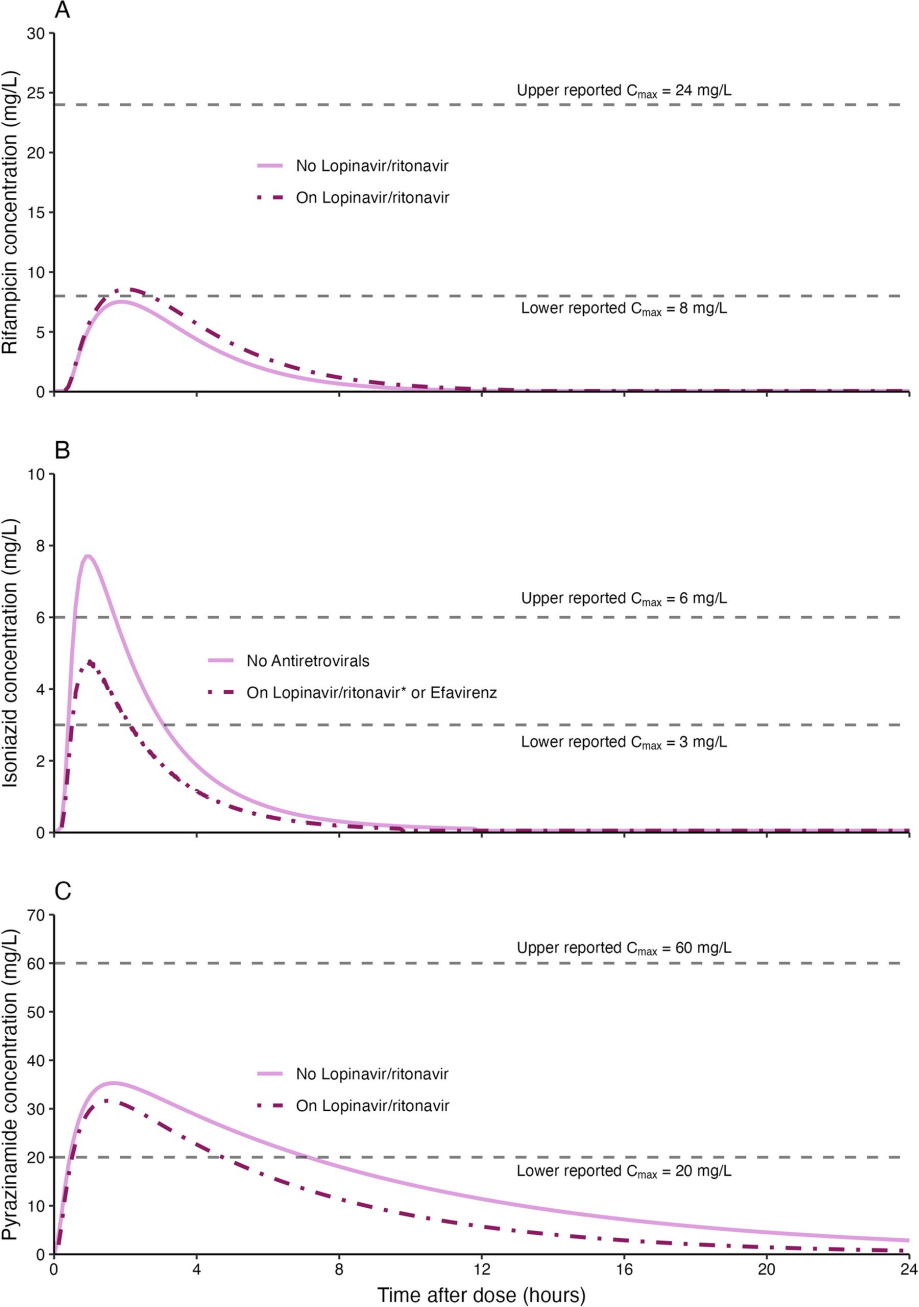
Concentration versus time plots for simulated steady-state drug concentrations. *When taken at the same time. Simulations of children weighing 14 kg on first-line tuberculosis drugs with or without antiretrovirals. Plot A shows the rifampicin concentrations achieved by a child on lopinavir/ritonavir versus when they are not on lopinavir/ritonavir. Plot B shows the isoniazid concentrations achieved by a child on lopinavir/ritonavir taken at the same time with isoniazid or on efavirenz, versus when they are not on any antiretrovirals. Plot C shows the pyrazinamide concentrations achieved by a child on lopinavir/ritonavir versus when they are not on lopinavir/ritonavir. The grey dashed lines represent the lower and upper boundaries of the maximum concentrations (C_max_) range reported in adults. The children were dosed according to 2010 WHO-recommended pediatric tuberculosis doses. Figures D, E, F, and G in S1 Text show VPCs stratified by study and lopinavir/ritonavir. VPC, visual predictive check; WHO, World Health Organization.

**Fig 4:**
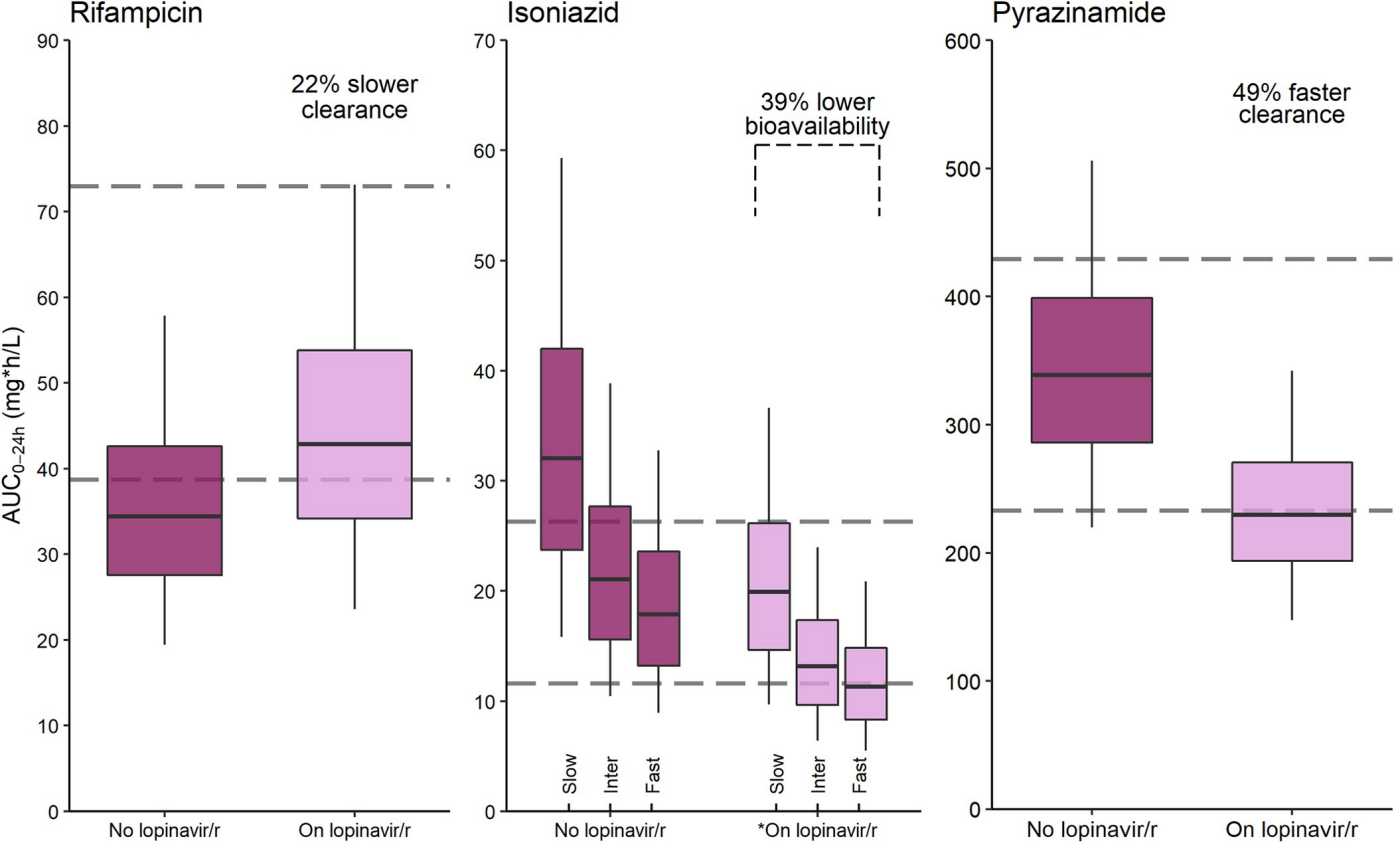
Simulated tuberculosis drug exposures achieved in children on lopinavir/ritonavir vs. those not on lopinavir/ritonavir. *When taken at the same time. Simulated rifampicin, isoniazid, and pyrazinamide AUC from time zero to 24 h in steady state (AUC_0-24h_) from concentrations achieved with the WHO-recommended weight-based dosing with the existing fixed dose combination formulation for approximately 65,000 patients weighing 3 to 36.9 kg. The pink boxes represent patients on lopinavir/ritonavir while the maroon boxes represent patients not on lopinavir/ritonavir. The difference in pharmacokinetic parameters between the 2 groups is indicated in the text above each plot. The isoniazid box plots are further stratified into fast, intermediate, and slow NAT2 acetylator-status. The gray horizontal dashed lines represent the upper and lower boundaries of the reported adult median AUC_0-24h_ ranges (72.9 to 38.7, 26.3 to 11.6, and 429 to 233 mg*h/L) for rifampicin, isoniazid, and pyrazinamide, respectively. The boxes indicate the interquartile range, while the whiskers denote the 2.5th and the 97.5th percentiles. AUC, area under the curve; NAT2, N-acetyl-2-transferase; WHO, World Health Organization.

We used final models to simulate area under concentration-time curve from 0 to 24 h (AUC_0-24h_) and maximum concentrations (C_max_) in an in silico representative population of 110,000 African pediatric patients (50% female) with uniformly distributed weight [22]. We compared simulated values with values reported in adults [5,23,24]. The following scenarios were investigated: (i) 2010 WHO-recommended pediatric tuberculosis weight-band (4.0–7.9, 8.0–11.9, 12.0–15.9, 16.0–24.9, and 25.0–36.9 kg) doses implemented with the available FDC (rifampicin/isoniazid/pyrazinamide 75 mg/50 mg/150 mg) (Fig 1 and Figure A in S1 Text); (ii) adding 75 mg or 150 mg tablet of rifampicin to top-up to 2010 WHO-recommended pediatric tuberculosis weight-band doses using the available FDC (Fig 2); (iii) first-line tuberculosis treatment doses using the FDC with ratios (rifampicin/isoniazid/pyrazinamide 120/30/135 mg) and weight-bands (3<6, 6<13, 13<20, 20<25 kg) recommended by Denti and colleagues (Table A in S1 Text and Figure B in S1 Text) [13]; (iv) 2010 WHO-recommended pediatric tuberculosis doses using weight-bands commonly used for HIV (3<6, 6<10, 10<15, 15<20, 20<25, 25<30, 30<40 kg) [25] (Text B in S1 Text, Tables B and C in S1 Text, Text C in S1 Text, and Figure C in S1 Text); and (v) compared participants on lopinavir/ritonavir versus those not on lopinavir/ritonavir (Figs 3 and 4).

## Results

### Study population

Data from 387 children (116 SHINE, 91 DNDi, and 180 DATiC) were available for analysis. The median (range) age and weight were 2.2 (0.2 to 15.0) years and 10.9 (3.2 to 59.3) kg, respectively. Approximately 47% were female and 151 (39.0%) were living with HIV, of whom 95.4% were on ART. NAT2 acetylator-status was available in 142 (35.8%) children from DATiC, 35 (25%), 81 (57%), and 26 (18%) were slow, intermediate, and fast-acetylators, respectively. Participant characteristics are summarized in Table 2. Overall, 2,625, 1,996, and 1,865 concentrations for rifampicin, isoniazid, and pyrazinamide, respectively, were available for analysis. Of these, 21%, 19.5%, and 2.8% of these were below the LLOQ for rifampicin, isoniazid, and pyrazinamide, respectively.

**Table 2:** Patient characteristics stratified by study.

			SHINE[11] (*N* = 116)	DNDI[12] (*N* = 91)	DATIC[13] (*N* = 180)	Total (*N* = 387)
**Weight (kg)**	Median (Min–Max)		14.2 (5.5–59.3)	8.8 (3.9–14.9)	10.9 (3.2–28.8)	10.9 (3.2–59.3)
**Height (cm)**	Median (Min–Max)		95.5 (53.7–176)	73 (55–101)	80.6 (49.9–135)	81.0 (49.9–176)
**Sex**: *n* (%)						
Female			55/116 (47.4%)	52/91 (57.1%)	74/180 (41.1%)	181/387 (46.8%)
Male			61/116 (52.6%)	39/91 (42.9%)	106/180 (58.9%)	206/387 (53.2%)
**Age (years)**	Median (Min–Max)		4.3 (0.3–15)	1.6 (0.3–6.8)	2 (0.2–11.9)	2.2 (0.2–15)
**Drug administration method**: *n* (%)						
Unknown			0 (0%)	91/91 (100%)	0 (0%)	91/387 (23.5%)
Whole			51/116 (44%)	0 (0%)	43/180 (23.9%)	94/387 (24.3%)
Dispersed or crushed			63/116 (54.3%)	0 (0%)	59/180 (32.8%)	122/387 (31.5%)
Crushed tablet and syringe			0 (0%)	0 (0%)	38/180 (21.1%)	38/387 (9.8%)
Crushed tablet and nasogastric tube			2/116 (1.7%)	0 (0%)	40/180 (22.2%)	42/387 (10.9%)
**Formulation**: *n* (%)						
pHRZ (McLeod’s)*			55/116 (47.4%)	N/A	N/A	55/387 (14.2%)
pHR (McLeod’s)**			38/116 (32.8%)	N/A	N/A	38/387 (9.8%)
HRZ (McLeod’s)***			14/116 (12.1%)	N/A	N/A	14/387 (3.6%)
HR (McLeod’s)#			9/116 (7.8%)	N/A	N/A	9/387 (2.3%)
RH_60–60 (Rimactazid, Sandoz, RSA)##			N/A	91/91 (100%)	N/A	91/387 (23.5%)
R (EREMFAT)###			N/A	N/A	115/180 (63.9%)	115/387 (29.7%)
R-CIN^			N/A	N/A	59/180 (32.8%)	59/387 (15.2%)
RH (Rimactazid, Norvatis, India)^^^^			N/A	N/A	6/180 (3.3%)	6/387 (1.6%)
**HIV Status**: *n* (%)						
Negative			81/116 (69.8%)	0 (0%)	150/180 (83.3%)	231/387 (59.7%)
Positive			35/116 (30.2%)	91/91 (100%)	25/180 (13.9%)	151/387 (39%)
Exposed through mum			0 (0%)	0 (0%)	4/180 (2.2%)	4/387 (1%)
Missing			0 (0%)	0 (0%)	1/180 (0.6%)	1/387 (0.3%)
**Antiretroviral therapy**: *n* (%)						
Not yet started			2/116 (5.7%)	0 (0%)	5/180 (17.2%)	7/387 (4.5%)
Efavirenz			15/116 (42.9%)	0 (0%)	6/180 (20.8%)	21/387 (13.5%)
Lopinavir/ritonavir			18/116 (51.4%)	91/91 (100%)	13/180 (44.8%)	122/387 (78.7%)
Nevirapine			0 (0%)	0 (0%)	5/180 (17.2%)	5/387 (3.3%)
**Rifampicin dose (mg/kg)** ^**^^^**^		Median (Min–Max)	14.4 (8.6–19.7)	12.5 (9.2–18.2)	15.5 (10.1–20.5)	14.0 (8.6–20.5)
**Isoniazid dose (mg/kg)**		Median (Min–Max)	9.6 (3.9–17.6)	12.4 (9.2–18.2)	12.0 (10.0–21.5)	11.7 (3.9–21.5)
**Pyrazinamide dose (mg/kg)**		Median (Min–Max)	28.2 (18.8–37.5)	29.8 (19.2–43.1)	34.1 (23.4–41.7)	32.1 (18.8–43.1)

*Pediatric formulation containing 75 mg rifampicin, 50 mg isoniazid, and 150 mg pyrazinamide.

**Pediatric formulation containing 75 mg rifampicin and 50 mg isoniazid.

***Adult formulation containing 150 mg rifampicin, 75 mg isoniazid, and 400 mg pyrazinamide.

^#^Adult formulation containing 150 mg rifampicin and 75 mg isoniazid.

^##^Pediatric formulation containing 60 mg rifampicin and 60 mg isoniazid.

^###^EREMFAT rifampicin only (20 mg/ml) granulates for suspension.

^^^R-Cin rifampicin only 100 mg/5 ml suspension.

^^^^Rimactazid pediatric formulation containing 100 mg rifampicin and 50 mg isoniazid.

^^^^^Following 2010 WHO pediatric tuberculosis dosing guidelines. N/A = not applicable.

DNDi, Drugs for Neglected Diseases initiative; HIV, human immunodeficiency virus; WHO, World Health Organization.

### Population pharmacokinetic analysis

A one-compartment disposition model with saturable clearance from a liver compartment and transit absorption best described rifampicin data. A two-compartment disposition model with first-order elimination and transit absorption fit isoniazid data best. A one-compartment disposition model with first-order elimination and absorption with lag time best described pyrazinamide data. Final parameter estimates with 95% confidence intervals (CIs) are shown in Table 3.

**Table 3:** Final model parameters for rifampicin, isoniazid, and pyrazinamide.

**Typical values (95% CI)** **+**			
**Parameter (units)**	Rifampicin	Isoniazid	Pyrazinamide
**Clearance–CL (L/h)** ^**1**^	52.5 (46.7, 59.0)		1.27 (1.19, 1.33)
Slow Intermediate Fast	-	4.01 (3.61, 4.61)* 6.16 (5.46, 6.85)** 7.06 (6.33, 7.91)***	-
**Central volume of distribution—V (L)**1	13.7 (12.7, 14.9)	13.8 (12.9, 14.8)	10.8 (10.4, 11.2)
**Intercompartmental clearance—Q (L/h)**1	-	0.509 (0.438, 0.584)	-
**Peripheral volume of distribution (L)**1	-	5.73 (4.85, 6.71)	-
**Bioavailability–F**	1 FIXED	1 FIXED	1 FIXED
**Mean absorption transit time—MTT (h)**	0.515 (0.439, 0.603)	0.300 (0.245, 0.362)	-
**Number of absorption transit compartments—NN (*n*)**	18.5 (15.4, 22.3)	10 FIXED	-
**Lag time (h)**	-	-	0.154 (0.098, 0.218)
**First-order absorption rate constant—Ka (1/h)**	2.72 (1.72, 2.29)	3.56 (3.03, 3.79)	1.89 (1.65, 2.25)
**Hepatic blood flow—Q**_**H**_ **(L/h)**^**2**^	90 FIXED	-	-
**Hepatic volume—V**_**H**_ **(L)**^**2**^	1 FIXED	-	-
**Fraction of unbound drug—F**_**u**_	0.2 FIXED	-	-
**Michaelis–Menten rate constant K**_**m**_ **—(mg/L)**	9.39 (7.96, 11.1)	-	-
**Formulation on F (%)** ^**3**^	−63.3 (−57.4, −69.2); *p* < 0.001	−33.9 (−20.0, −46.5); *p* < 0.001	-
**Crushing tablets on Ka (%)** ^**4**^	-	-	**+**98.0 (**+**64.0, **+**137); *p* < 0.001
**Lopinavir/ritonavir on CL (%)**	−22.3 (−12.8, −28.0); *p* < 0.001	-	**+**48.5 (**+**39.4, **+**57.7); *p* < 0.001
**Lopinavir/ritonavir on F (if ingested simultaneously) (%)**	-	−39.2 (−32.1, −45.2); *p* < 0.001	-
**Efavirenz on F (%)**	-	−37.2 (−21.8, −51.9); *p* < 0.001	-
**Yesterday’s F (%)**	-	-	−36.6 (−29.3, −42); *p* < 0.001
**Study on F (%)** ^**5**^	-	-	−27.2 (−20.9, −34.1); *p* < 0.001
**Maturation of CL**			
Post-menstrual age at 50% adult CL (months)^6^	11.0 (9.90, 12.3); *p* < 0.001	10.9 (10.1, 11.7); *p* < 0.001	11.5 (10.2, 12.2); *p* < 0.001
Gamma (ƴ- shape of sigmoidal maturation function)	3.25 (2.72, 3.89); *p* < 0.001	3.31 (2.86, 3.95); *p* < 0.001	3.42 (2.29, 5.11); *p* < 0.001
**Age on F**			
F at Birth (%)	48.9 (36.0, 61.8); *p* < 0.001	64.5 (52.1, 78.9); *p* < 0.001	-
Age at adult F (months)	27.7 (20.7, 37.0); *p* < 0.001	26.5 (14.2, 41.0); *p* < 0.001	-
**Lab_Scaling (fold change)** ^**7**^	1.22 (0.933, 1.47); *p* < 0.001	-	1.34 (1.28, 1.42); *p* < 0.001
**BSV (%)**			
V	-	21.5 (17.0, 25.1)	-
CL	34.9 (30.1, 39.2)	27.3 (23.2, 31.5)	22.0 (20.4, 24.5)
**BOV (%)**			
F	35.4 (32.9, 37.6)	44.8 (40.9, 52.0)	29.8 (28.0, 33.2)
MTT	42.1 (33.1, 49.4)	81.2 (70.3, 94.6)	-
Ka	162 (143, 180)	82.0 (65.0, 98.2)	71.3 (61.8, 82.6)
Lag time	-	-	91.7 (70.5, 121.6)
**Study or site on BOV on F (fold change)**	-	1.76 (1.45, 2.11); *p* < 0.001ⱡ	2.74 (2.18, 3.32); *p* < 0.001ⱡⱡ
**Residual unexplained error**			
Proportional error (%)	24.1 (23.3, 24.9)	14.2 (13.5, 14.9)	7.21 (7.15, 8.03)
Additive error (mg/L)	0.023 FIXED	0.0195 FIXED	0.0400 FIXED
Pre-dose additive error (mg/L)^8^	-	-	1.10 (0.885, 1.25); *p* < 0.001

^1^All clearances and volumes of distribution were allometrically scaled and the typical values reported here refer to a child weighing 14 kg. For rifampicin, the value reported is the intrinsic clearance.

^2^These values are for an adult male of 70 kg. Then, it was allometrically scaled by weight.

^3^This relationship was for the R-Cin formulation for rifampicin and the McLeod’s adult formulation for isoniazid.

^4^Those that had their tablets crushed had a faster absorption.

^5^This was a scaling factor for the DNDi study.

^6^Post-natal age + gestation.

^7^This was a factor scaling the samples that were assayed in India within the SHINE study.

^8^Extra additive error on the pre-dose samples.

^+^Based on SIR.

*Proportion of slow NAT2 acetylators fixed to 44%.

**Proportion of intermediate NAT2 acetylators fixed to 42%.

***Proportion of fast NAT2 acetylators fixed to 14%.

^ⱡ^This relationship was for the DNDi study.

^ⱡⱡ^This was a scaling factor for the Tygerberg site in the DNDi study.

BOV, between-occasion variability; BSV, between-subject variability; CI, confidence interval; DNDi, Drugs for Neglected Diseases initiative; NAT2, N-acetyltransferase 2; SIR, sampling importance resampling.

**Fig 5:**
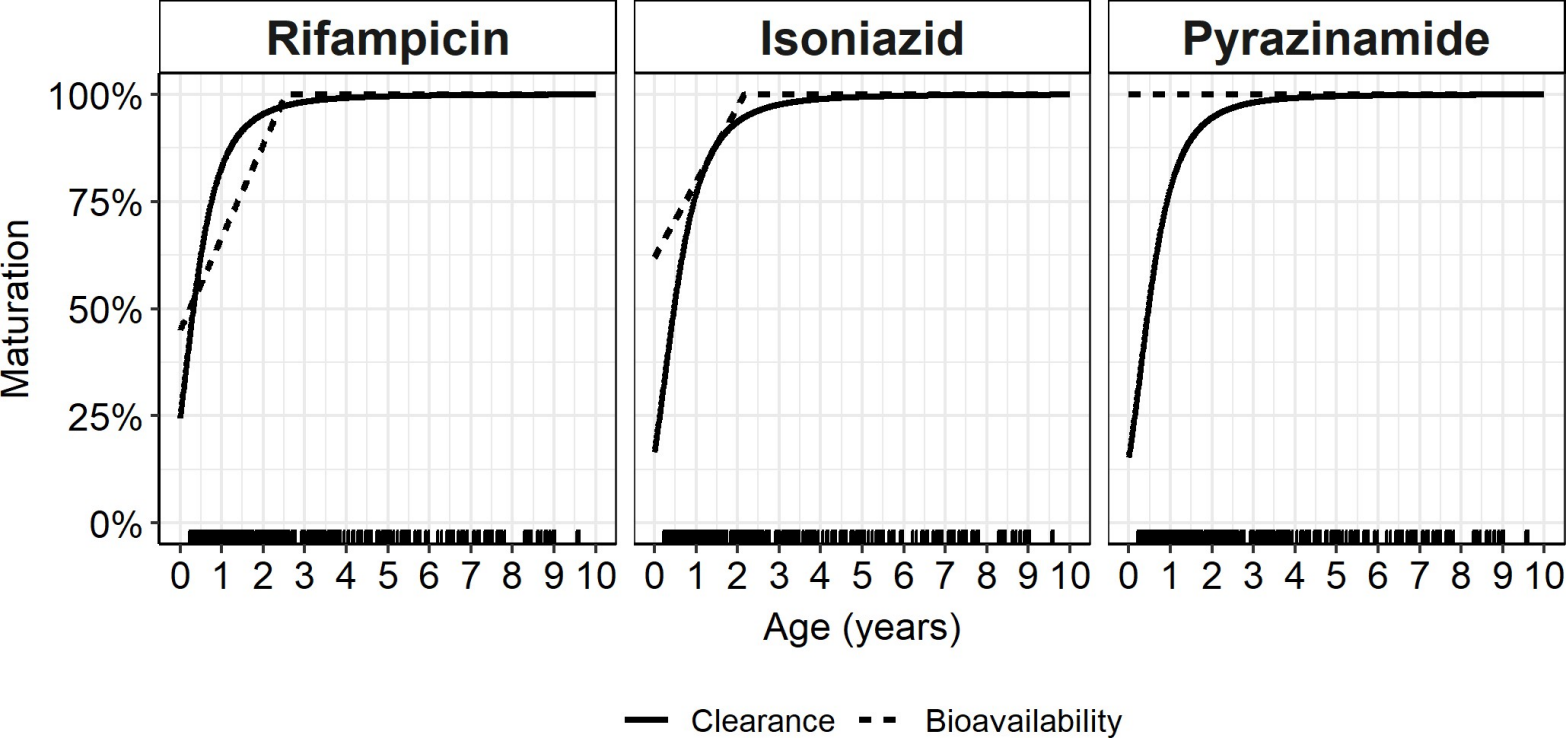
Plot of maturation of clearance and bioavailability. Plot of maturation of clearance (solid line) and bioavailability (dashed line) of rifampicin, isoniazid, and pyrazinamide. The vertical lines at the bottom indicate the ages for which we had observations.

Allometric scaling of clearance and volume improved the fit for all 3 drugs. The exponents of clearance and volume were fixed, and there were therefore no additional degrees of freedom, hence, no *p*-values could be computed. Fat-free mass (FFM)-based scaling was better than weight for pyrazinamide (change in objective function value (dOFV) = 3.18), and weight was preferred for rifampicin (dOFV = 1.42). For isoniazid, as there was a very small difference between FFM and weight (dOFV = 0.390), we maintained the weight. The fit for all drugs further improved after we added maturation of clearance (dOFV = 34.8; *p* < 0.001, 42.9; *p* < 0.001, and 14.8; *p* < 0.001), for rifampicin, isoniazid, and pyrazinamide, respectively. Bioavailability increased linearly with age, until adult bioavailability was reached at 27.7 months (95% CI [20.7 months, 37.0 months]; dOFV = 38.3; *p* < 0.001) and 26.5 months (95% CI [14.2 months, 41.0 months]; dOFV = 24.2; *p* < 0.001) months for rifampicin and isoniazid, respectively. Pyrazinamide bioavailability was unchanged with age. Fig 5 shows the change in clearance and bioavailability from birth for all 3 drugs.

In children on lopinavir/ritonavir, rifampicin clearance was 22% (95% CI [13%, 28%]; dOFV = 19.0; *p* < 0.001) lower, while pyrazinamide clearance was increased by 49% (95% CI [39%, 57%]; dOFV = 130.0; *p* < 0.001) compared to all other children in the analysis. Isoniazid bioavailability was 39% (95% CI [32%, 45%], dOFV = 50.0; *p* < 0.001) lower in children taking their lopinavir/ritonavir dose at the same time as isoniazid (but not when the administration was at least 2 h apart) and 37% (95% CI [22%, 52%]; dOFV = 17.0; *p* < 0.001) lower in children receiving efavirenz, compared to all other children in the analysis. Figure D in S1 Text panels 1, 2, and 3 show the model fit for children who are HIV-, HIV+ not yet on ART, and HIV+ who took lopinavir/ritonavir 2 h before isoniazid, while panels 4 and 5 show the model fit for children with HIV who took lopinavir/ritonavir at the same time as isoniazid and those on efavirenz, respectively. The bioavailability in the first 3 strata is similar, negating the influence of HIV on the pharmacokinetics of isoniazid, the last 2 strata have lower exposures, showing that the difference is present due to lopinavir/ritonavir taken at the same time with isoniazid and efavirenz co-treatment.

The R-Cin rifampicin-only formulation taken in the DATiC study had 63% (95% CI [57%, 69%], dOFV = 20.0; *p* < 0.001) lower bioavailability compared to other rifampicin formulations pooled together. The McLeod’s adult drug FDC formulations used in SHINE had 34% (95% CI [20%, 47%]; dOFV = 18.0; *p* < 0.001) lower isoniazid bioavailability than other isoniazid formulations pooled together. Table 3 shows parameter estimates and covariates for all 3 drugs and Figures D, E, F, and G in S1 Text show the final model fit for all drugs.

### Simulation results

AUC and C_max_ simulations with 2010 WHO-recommended tuberculosis weight-band dosing implemented with available FDC had median rifampicin, isoniazid, and pyrazinamide AUCs and C_max_ above reported adult exposures for children younger than 3 months.

For children older than 3 months, median rifampicin AUCs were lower than reported adult AUCs in all weight-bands except the 16.0 to 24.9 kg weight-band. For isoniazid, median AUCs were above observed adult AUCs in children weighing 12.0 to 24.9 kg. Median pyrazinamide AUCs were within observed adult AUCs in all weight-bands (Fig 1 and Figure A in S1 Text).

Simulations with additional rifampicin doses of 75 mg and 150 mg in children weighing <25 kg and >25 kg respectively, to top-up 2010 WHO-recommended weight-based tuberculosis doses, showed improved rifampicin exposures in children in all weight bands, leading to median exposures similar to those observed in adults (Fig 2).

Simulations with 2010 WHO-recommended tuberculosis doses implemented with the available FDC and weight-bands commonly used in HIV showed improved rifampicin exposures in children weighing 6 to 8, 10 to 12, and 14 to 16 kg (Tables B and C in S1 Text and Figure C in S1 Text), compared to using 2010 WHO-recommended weight-bands. Simulations with the new FDC and weight-bands suggested by Denti and colleagues showed the optimal exposures (Table A in S1 Text and Figure B in S1 Text) [13].

Simulations of scenarios with and without lopinavir/ritonavir showed increased rifampicin exposures in the former. The exposure-reducing impact of taking lopinavir/ritonavir at the same time with isoniazid was most pronounced in fast-acetylators. Lopinavir/ritonavir reduced pyrazinamide exposures (Figs 3 and 4).

## Discussion

In this model-based pooled individual patient data pharmacokinetic analysis, we combined data from 3 studies of children on the 2010 WHO-recommended first-line tuberculosis treatment. This gave us a strong and unique dataset that allowed us to develop robust population pharmacokinetic models to evaluate the WHO dosing guidelines and explore the associations of HIV, ART, drug formulation, age, and body size with the pharmacokinetics of first-line tuberculosis drugs. We observed no association of HIV with the pharmacokinetics of rifampicin, isoniazid, or pyrazinamide. We found children on lopinavir/ritonavir had higher rifampicin, lower pyrazinamide, and lower isoniazid (when isoniazid and lopinavir/ritonavir were ingested simultaneously) exposures. Children on efavirenz also had lower isoniazid exposure. Two formulations led to further lower rifampicin and isoniazid exposures: the R-Cin rifampicin formulation and the Mcleod’s adult FDC formulation, respectively.

There were significant drug–drug interactions between first-line tuberculosis drugs and lopinavir/ritonavir. There was 22% reduction in rifampicin clearance in children on lopinavir/ritonavir compared to other children on rifampicin without lopinavir/ritonavir. Ritonavir inhibits intestinal and hepatic p-glycoprotein and also organic anion-transporting polypeptides 1 and 3 efflux transporters in the liver, possibly resulting in reduced excretion of rifampicin into bile [26]. Our findings are consistent with 2 studies of adults on steady-state rifampicin where 5 patients coinfected with HIV and tuberculosis were on lopinavir/ritonavir and 10 healthy volunteers were on atazanavir [7,27]. Future studies considering higher rifampicin doses should pay attention to children on lopinavir/ritonavir co-treatment, as they may exhibit higher than desired rifampicin concentrations.

In children on lopinavir/ritonavir, pyrazinamide exposure was 32.2% lower due to increased clearance. In vitro evidence suggests this might be attributed to ritonavir’s activation of xanthine oxidase [28], the enzyme which metabolizes pyrazinamide [29]. To our knowledge, this association has not been reported previously [7]. It is possible that we were able to detect it because 80% of the children on lopinavir/ritonavir were super-boosted by additional ritonavir (lopinavir/ritonavir 4:4 rather than 4:1) in the presence of rifampicin. Because pyrazinamide is essential in reducing tuberculosis relapse risk [30], the potential consequences of the reduced pyrazinamide exposure should be considered in designing ART regimens for children with tuberculosis.

When isoniazid and lopinavir/ritonavir were ingested simultaneously, lower isoniazid exposures were observed due to 39% reduced isoniazid bioavailability. We hypothesize that this is due to the high fructose content (15.3% w/v) in Kaletra, the lopinavir/ritonavir formulation used in all 3 studies [31]. Rao and colleagues noted isoniazid condensation when mixed with different sugars [32,33]. We hypothesize that an interaction between fructose and isoniazid may have led to reduced isoniazid available for absorption. Children on isoniazid-containing tuberculosis treatment may benefit if ingestion of co-prescribed syrups is separated by at least 1 or 2 h from isoniazid.

The association of efavirenz with isoniazid pharmacokinetics has been described previously [6,34]. Although these studies reported an increased clearance of isoniazid in fast-acetylating adults, our finding of lower isoniazid exposure in children on efavirenz was independent of isoniazid acetylator-status. This is probably due to the small number (13.7%) of children on efavirenz in our study, and missing NAT2 acetylator-status information in some children, which limited our ability to detect the explanatory effect of acetylator-status. Additionally, in children in this analysis, efavirenz was administered within the same time frame as isoniazid. The association we found could be a result of pre-hepatic interactions between efavirenz and isoniazid, but we cannot make conclusions since we did not have a group which took efavirenz hours apart from isoniazid, to serve as a comparator.

Since the associations of efavirenz and lopinavir/ritonavir on isoniazid bioavailability were similar, we explored a possible HIV association. We had 7 children with HIV, not yet on ART, and we could separate the association of each ART and HIV. We found no difference in isoniazid bioavailability among those without HIV and those with HIV but ART-naive, ruling out the association of HIV with isoniazid bioavailability (Figure D in S1 Text) [7]. Similar results were found when repeating the same test for rifampicin and pyrazinamide, where HIV infection did not change their pharmacokinetics. Most previous studies evaluating the association of HIV with tuberculosis-drug pharmacokinetics in children did so in the presence of ART [14,35,36]; hence, it may have been impossible to distinguish whether any association was due to HIV or to ART. Two studies that recruited ART-naïve children reported that HIV reduced the exposure of rifampicin, pyrazinamide, and ethambutol [37,38]. However, both these studies used non-compartmental analysis, a methodology with limited ability to account for confounding factors and nonlinear covariates (like age and weight), so the reported association of HIV may have been confounded by dose and malnutrition, which likely overlapped with HIV in these studies. Our analysis used nonlinear mixed effect modeling, and we could better account for the nonlinear confounding-factors.

With regards to formulation, we confirmed 63% lower rifampicin bioavailability in the R-Cin formulation compared to other rifampicin formulations, as reported by Denti and colleagues [13]. This is worrying because it exacerbates the problem of rifampicin under-exposure, especially in children weighing less than 12 kg, whom we found to have very low rifampicin AUC compared to those reported in adults. We also confirmed previous findings of lower AUC of isoniazid in children on the adult formulation in SHINE [39]. Furthermore, in our analysis we were able to conclude that this reduction was not only due to the lower dose for children in the >25 kg weight-band (4 to 6 mg/kg versus 10 mg/kg) or allometry, since both factors were accounted for in the model. These results stress the importance of continued drug-quality monitoring and bioequivalence studies in intended populations.

Results from our simulations showed that the 2010 WHO-recommended weight-based dosing of first-line tuberculosis-drugs leads to suboptimal drug exposures because it does not account for young children’s maturation of clearance and does not completely account for the differences in body size between children and adults [40]. Of note, children under 3 months were predicted to have higher median AUC_0-24h_ and C_max_ for all 3 drugs, when compared to observed adult exposures. Although we did not have many children under 3 months, our results are consistent with previous reports [13]. Whether higher exposures in this age-group lead to drug-induced toxicities remains to be investigated. Moreover, the safe and effective range for these medicines could be wider than the reported observed adult median values. As a short-term precautionary measure, we recommend monitoring for hepatic toxicity symptoms in children younger than 3 months during first-line tuberculosis treatment. In children older than 3 months, median rifampicin exposures were low in most weight-bands, confirming previous findings [13,41]. We recommend higher rifampicin doses in children older than 3 months. As a quick, short-term solution, we suggest topping up rifampicin doses with 75 mg and 150 mg rifampicin only tablets in children weighing <25 kg and >25 kg, respectively. A rifampicin top-up would be feasible to implement short-term because the existing 150 mg single drug rifampicin formulations could be used (half a tablet for children weighing <25 kg) and no new FDC would have to be manufactured. As a long-term solution, we recommend adopting the doses, FDC and weight-bands recommended by Denti and colleagues [13] as shown in Figure B in S1 Text. Finally, if the WHO endorses harmonizing weight-bands across different diseases using weight bands commonly used in HIV (using 2010 WHO-recommended doses and currently available FDCs), the exposures achieved would be comparable with those achieved with the 2010 WHO-recommended weight-bands, with slightly increased exposures in children weighing 6 to 16 kg. With these weight bands, we also recommend supplementing rifampicin with 75 mg and 150 mg rifampicin only tablets in children weighing <25 kg and >25 kg, respectively. Using the HIV weight-bands would benefit HIV and tuberculosis coinfected patients and healthcare prescribers who would find it convenient to use the same weight-bands. Increased rifampicin doses could exacerbate drug–drug interactions and might warrant re-evaluation, but recent findings have shown that the additional induction effect is limited [41].

The strengths of our study are that firstly, we pooled 3 large studies conducted almost synchronously, all intensively sampled, and whose drug concentrations for the majority were assayed in the same laboratory, which substantially reduced impact of inaccuracy in drug quantification [42]. This large and unique dataset enabled us to develop robust models exploring and accounting for associations of age and body size, drug-formulation, HIV, and ART. Our analysis has an advantage over pooling individual summary measures, which accumulate errors and/or bias contained in the calculations derived differently for different studies [43]. The main limitation of our analysis was that, despite the huge similarities in the studies and consistency in the assay method across studies, there were small pharmacokinetic differences that had to be accounted for in the model. For example, all children in DNDi were on super-boosted lopinavir/ritonavir while only a few were on this ART regimen in other studies, different tuberculosis drug formulations were used across the studies, some children in the DATiC study were given lopinavir/ritonavir simultaneously with isoniazid while others were not. This is likely the reason why we still had study and site-specific differences that could not be explained otherwise. For this reason, future studies need to independently confirm our findings, since some unaccounted confounders cannot be ruled out. This also calls for standardization of pediatric pharmacokinetic studies and collection of accurate information about drug formulation, method, and timing of administration of all drugs and food intake. This would make future pooling of studies easier. Finally, having NAT2 genotype information in the whole population may have reduced this unexplained study variability and further reduced risks of confounding by study.

In conclusion, the main finding of our study is that children older than 3 months on the 2010 WHO-recommended dosing achieve lower rifampicin exposures than adults. On the other hand, rifampicin, isoniazid, and pyrazinamide drug concentrations may be too high in children younger than 3 months, for whom we recommend toxicity monitoring. A further revision to WHO pediatric tuberculosis dosing guidelines could address these issues. Increasing rifampicin doses would be a good start and can be quickly achieved by adding a rifampicin tablet. If harmonized weight bands are adopted, then higher rifampicin doses would still apply. If the guidelines were updated completely, we would recommend the optimized FDC formulation and optimized weight-bands recommended by Denti and colleagues [13]. In terms of associations of HIV and ART, we report that drug–drug interactions with ART, and not HIV infection per se [14,35,36], may alter tuberculosis-drug concentrations. As a precaution, we recommend that where tuberculosis-drugs and sugar-containing ART formulations are concurrently indicated, if possible, they should be taken at least 2 h apart to avoid possible prehepatic interactions. Finally, we advocate stricter monitoring of tuberculosis drug formulations.

## Supporting information

S1 Text Supplementary material. Figure A. Simulated drug exposures after administration of WHO recommended doses and weight bands. Text A. DATiC recommended optimized weight bands and fixed dose combination tablets. Table A. DATiC recommended optimized weight bands and fixed dose combination tablet compared with WHO recommended doses and weight bands. Figure B. Simulated drug exposures achieved after administration of DATiC recommend fixed dose combination tablets and weight bands. Text B. Harmonized weight bands. Table B. Number of tablets to be taken once daily based on WHO-recommended doses, the current fixed-dose combination formulation* and harmonized weight bands. Table C. Number of tablets to be taken once daily by children weighing ≥25 kg based on WHO recommended adult doses, the current adult fixed-dose combination formulation and harmonized weight bands. Text C. Harmonized weight band simulation results. Figure C. Simulated drug exposures achieved after administration of WHO-recommended doses with harmonized weight bands (similar to those used in HIV). Figure D. Visual predictive check of isoniazid concentration versus time after dose, stratified by HIV status and antiretroviral therapy. Figure E. Visual predictive check of drug concentration versus time after dose, stratified by study. Figure F. Visual predictive check of rifampicin drug concentration versus time after dose, stratified by lopinavir/ritonavir. Figure G. Visual predictive check of pyrazinamide drug concentration versus time after dose, stratified by lopinavir/ritonavir.
(DOCX)

## Abbreviations

ART: antiretroviral therapy
BOV: between-occasion variability
BSV: between-subject variability
CI: confidence interval
DNDi: Drugs for Neglected Diseases initiative
FDC: fixed-dose combination
FFM: fat-free mass
FOCE-I: first-order conditional estimation with interaction
HIV: human immunodeficiency virus
LLOQ: lower limit of quantification
LOD: limit of detection
NAT2: N-acetyltransferase 2
SAEM: stochastic approximation expectation-maximization
SIR: sampling importance resampling
UCT: University of Cape Town
VPC: visual predictive check
WHO: World Health Organization
