# Supplementary material for: Evaluating pediatric tuberculosis dosing guidelines: A model-based individual data pooled analysis
Source: PLoS Med. 2023 Nov 21;20(11):e1004303. doi: 10.1371/journal.pmed.1004303 (PMC10662720; doi:10.1371/journal.pmed.1004303)
Supplement: S1 Text — Figure A. Simulated drug exposures after administration of WHO recommended doses and weight bands. Text A. DATiC recommended optimized weight bands and fixed dose combination tablets. Table A. DATiC recommended optimized weight bands and fixed dose combination tablet compared with WHO recommended doses and weight bands. Figure B. Simulated drug exposures achieved after administration of DATiC recommend fixed dose combination tablets and weight bands. Text B. Harmonized weight bands. Table B. Number of tablets to be taken once daily based on WHO-recommended doses, the current fixed-dose combination formulation* and harmonized weight bands. Table C. Number of tablets to be taken once daily by children weighing ≥25 kg based on WHO recommended adult doses, the current adult fixed-dose combination formulation and harmonized weight bands. Text C. Harmonized weight band simulation results. Figure C. Simulated drug exposures achieved after administration of WHO-recommended doses with harmonized weight bands (similar to those used in HIV). Figure D. Visual predictive check of isoniazid concentration versus time after dose, stratified by HIV status and antiretroviral therapy. Figure E. Visual predictive check of drug concentration versus time after dose, stratified by study. Figure F. Visual predictive check of rifampicin drug concentration versus time after dose, stratified by lopinavir/ritonavir. Figure G. Visual predictive check of pyrazinamide drug concentration versus time after dose, stratified by lopinavir/ritonavir. (DOCX) [file pmed.1004303.s001.docx]

**Supplementary material**

Evaluating pediatric tuberculosis dosing guidelines: A model-based individual data pooled analysis.

Lufina Tsirizani Galileya^1,2^, Roeland E. Wasmann^1^, Chishala Chabala^1,3,4^, Helena Rabie^5^, Janice Lee^6^, Irene Njahira Mukui^6^, Anneke Hesseling^7^, Heather Zar^8^, Rob Aarnoutse^9^, Anna Turkova^10^, Diana Gibb^10^, Mark F. Cotton^5^, Helen McIlleron^1,11^, Paolo Denti^1*^

**AFFILIATIONS:**

1. Division of Clinical Pharmacology, Department of Medicine, University of Cape Town, Cape Town, South Africa.

2. Training and Research Unit of Excellence, Kamuzu University of Health Sciences, Blantyre, Malawi.

3. Department of Pediatrics, University of Zambia, School of Medicine, Lusaka, Zambia.

4. University Teaching Hospitals-children’s Hospital, Lusaka, Zambia.

5. Department of Pediatrics and Child Health and Family Center for Research with Ubuntu, Stellenbosch University, Cape Town, South Africa.

6. Drugs for Neglected Diseases initiative, Geneva, Switzerland.

7. Desmond Tutu TB Centre, Department of Pediatrics and Child Health, Faculty of Medicine and Health Sciences, Stellenbosch University, Cape Town, South Africa.

8. Department of Pediatrics and Child Health, Red Cross War Memorial Children’s Hospital, and SA-MRC Unit on Child & Adolescent Health, University of Cape Town, Cape Town, South Africa.

﻿9. Radboud University Medical Center, Nijmegen, The Netherlands

10. Medical Research Council Clinical Trials Unit at University College London, Institute of Clinical Trials and Methodology, London, United Kingdom.

11. Wellcome Centre for Infectious Diseases Research in Africa (CIDRI-Africa), Institute of Infectious Disease and Molecular Medicine, University of Cape Town, Cape Town, South Africa.

***** [paolo.denti@uct.ac.za](mailto:paolo.denti@uct.ac.za)

# **Additional figures**

**WHO recommended dosing and weight bands with weight stratified per kilogram.**

### Figure A: Simulated drug exposures after administration of WHO recommended doses and weight bands.

**
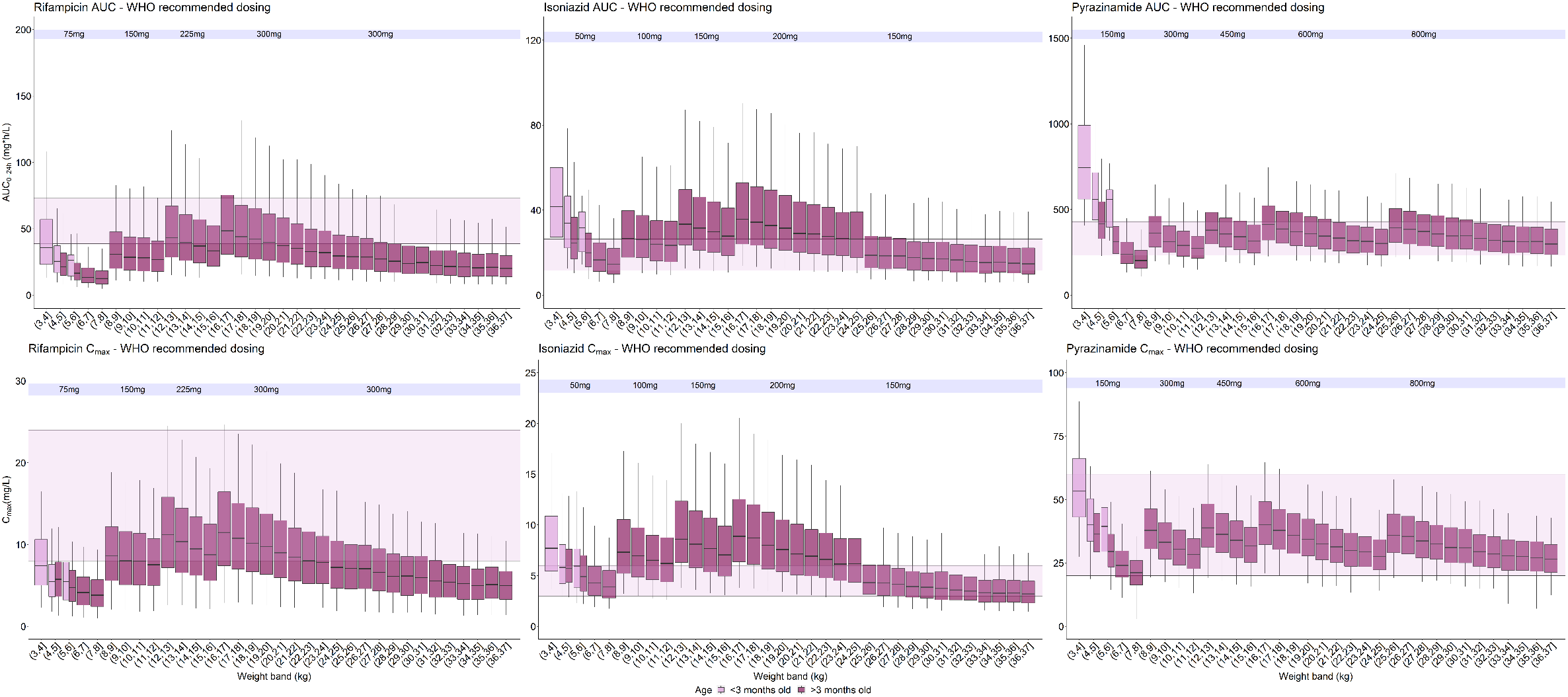

Figure A:** Simulated rifampicin, isoniazid and pyrazinamide AUC from time zero to 24 h (AUC_0–24_) and maximum concentrations (C_max_) versus body weight, with concentrations achieved with the (World Health Organization) (WHO)-recommended weight-based dosing and the existing fixed dose combination formulation[1], after adjusting for age and weight. The plum boxes represent patients < 3 months while the maroon boxes represent patients ≥ 3 months. The plum horizontal shaded area represents the reported adult median AUC_0-24_ ranges (38.7-72.9, 11.6-26.3 and 233-429 mg*h/L) and C_max_ ranges (8-24, 3-6, and 20-60 mg/L) for rifampicin, isoniazid and pyrazinamide respectively. The boxes indicate inter-quartile range, while the whiskers denote the 2.5^th^ and the 97.5^th^ percentiles.

# **Alternative dosing scenarios and weight bands**

**Text A: DATiC recommended optimized weight bands and fixed dose combination tablets.**

A recent study evaluating drug exposures of WHO-recommended first-line tuberculosis drugs in children suggested new weight-based doses and fixed dose combination (FDC) tablet (rifampicin/isoniazid/pyrazinamide 120 mg/30 mg/135 mg), (3<6kg, 6<13kg, 13<20, 20<25kg) to even out drug exposures across all weight bands [2]. We used our final models to run simulations with these suggested optimized doses and FDC. We found that median rifampicin, isoniazid, and pyrazinamide AUCs were within the reported median adult AUC ranges in almost all weight bands. Median rifampicin, isoniazid, and pyrazinamide C_max_ were within the reported median adult C_max_ ranges in all weight bands except children less than three months who had slightly lower median C_max_.

Table A**: DATiC recommended optimized weight bands and fixed dose combination tablet compared with WHO recommended doses and weight bands.**

|  | **WHO recommended FDC and weight bands** | **Rifampicin mg/kg achieved with WHO-recommended FDC and weight bands** | **Isoniazid mg/kg achieved with WHO-recommended FDC and weight bands** | **Pyrazinamide mg/kg achieved with WHO-recommended FDC and weight bands** | **Optimized FDC* and weight bands** | **Rifampicin mg/kg achieved with Optimized FDC* and weight bands** | **Isoniazid mg/kg achieved with Optimized FDC* and weight bands** | **Pyrazinamide mg/kg achieved with Optimized FDC* and weight bands** |
| --- | --- | --- | --- | --- | --- | --- | --- | --- |
| Rifampicin, mg | 75 | - | - | - | 120 | - | - | - |
| Isoniazid, mg | 50 | - | - | - | 30 | - | - | - |
| Pyrazinamide, mg | 150 | - | - | - | 135 | - | - | - |
|  | | | | | | | | |
| 1^st^ weight band (1 tablet), kg | 4-8 | 9.3-18.8 | 6.3-12.5 | 18.8-37.5 | 3-6 | 20.0-40.0 | 5.0-10.0 | 22.5-45.0 |
| 2^nd^ weight band (2 tablets), kg | 8-12 | 12.5-18.8 | 8.3-12.5 | 25.0-37.5 | 6-13 | 18.5-40.0 | 4.6-10.0 | 20.8-45.0 |
| 3^rd^ weight band (3 tablets), kg | 12-16 | 14.1-18.8 | 9.4-12.5 | 28.1-37.5 | 13-20 | 18.0-27.7 | 4.5-6.9 | 20.3-31.2 |
| 4^th^ weight band (4 tablets), kg | 16-25 | 12.0-18.8 | 8.0-12.5 | 24.0-37.5 | 20-25 | 19.2-24.0 | 4.8-6.0 | 21.6-27.0 |

*Fixed dose combination tablet.

### Figure B: Simulated drug exposures achieved after administration of DATiC recommend fixed dose combination tablets and weight bands.

**
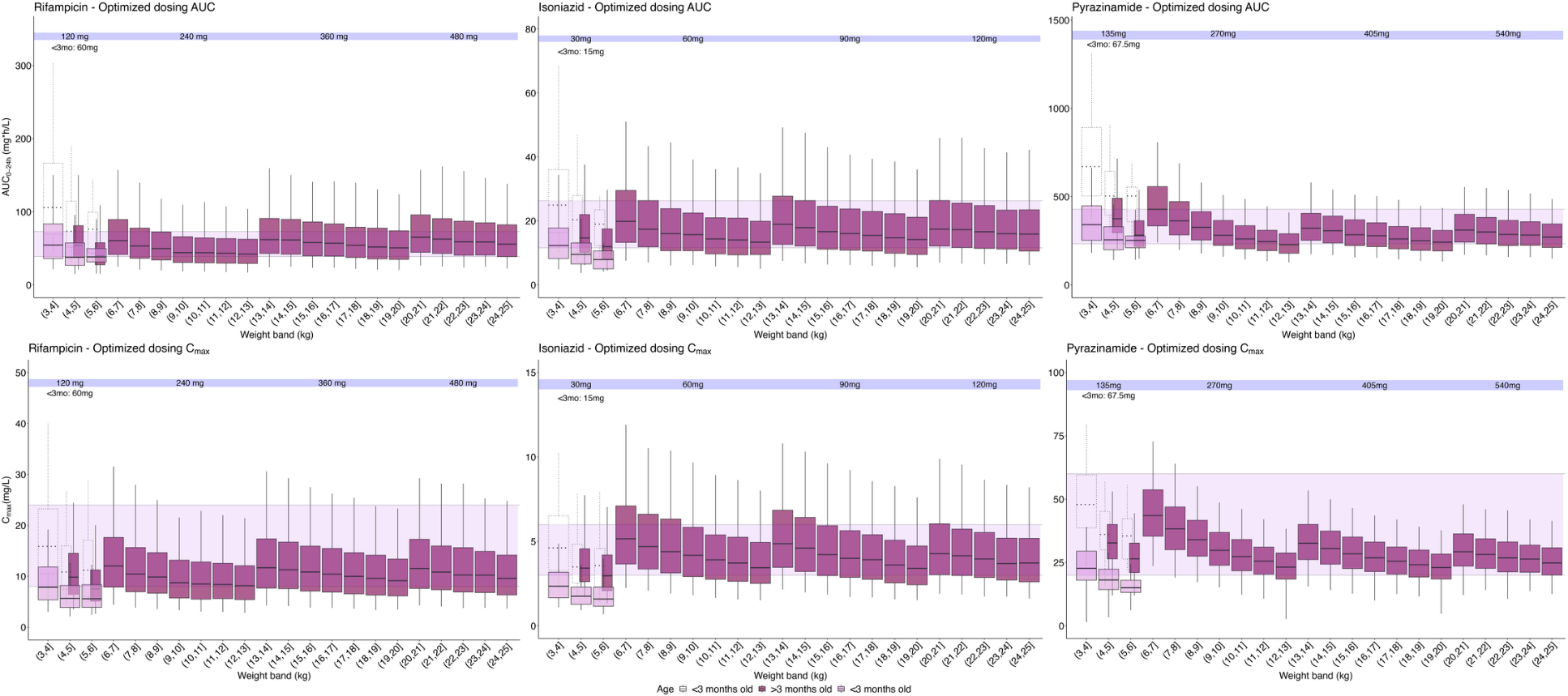
**

**Figure B:** Simulated rifampicin, isoniazid and pyrazinamide AUC from time zero to 24 h (AUC0–24) and maximum concentrations (Cmax) versus body weight, with concentrations achieved with the DATiC-recommended weight bands and new fixed dose combination (FDC) dose ratios, after accounting for age and weight. The plum boxes represent patients <3 months while the maroon boxes represent patients ≥3 months. The white dotted line box plots represent children <3 months on double the optimized dose recommended for that weight band. The plum horizontal shaded area represents the reported adult median AUC0-24 ranges (38.7-72.9, 11.6-26.3 and 233-429 mg*h/L) and Cmax ranges (8-24, 3-6, and 20-60 mg/L) for rifampicin, isoniazid and pyrazinamide respectively. The boxes indicate the inter-quartile range, while the whiskers denote the 2.5th and the 97.5th percentiles.

**Text B: Harmonized weight bands**

Background

In an effort to improve quality of care and ease prescription and administration of drugs for children with co-morbidities, harmonized weight bands across different therapeutic areas have been suggested as a solution. We used our models to evaluate first-line tuberculosis drug exposures when using weight bands commonly used for HIV. The current weight bands are as follows: 4.0-7.9kg, 8.0-11.9kg, 12.0-15.9kg, 16.0-25kg, 25.0-36.9kg. The harmonized weight bands and doses given are shown in supplementary Tables 2a and 2b.

Table B**: Number of tablets to be taken once daily based on World health organization (WHO) recommended doses, the current fixed dose combination formulation * and harmonized weight bands.**

| **Weight Bands** | Intensive Phase | | Continuation Phase |
| --- | --- | --- | --- |
|  | **75R, 50H, 150Z** | **100E** | **75R, 50H** |
| 3.0 – 5.9 kg | 1 | 1 | 1 |
| 6.0 – 9.9 kg | 2 | 2 | 2 |
| 10.0 – 14.9 kg | 3 | 3 | 3 |
| 15.0 – 19.9 kg | 4 | 4 | 4 |
| 20.0 – 24.9 kg | 4 | 4 | 4 |

*Rifampicin €:15mg/kg (range 10–20mg/kg) to a maximum dose 600mg/day; Isoniazid (H):10mg/kg (range 7–15mg/kg) to a maximum dose 300mg/day; Pyrazinamide (Z): 35mg/kg (30–40mg/kg); Ethambut€(E): 20mg/kg (15-25mg/kg).

Table C: **Number of tablets to be taken once daily by children weighing ≥ 25 kg** **based on World health organization (WHO) recommended adult doses, the current adult fixed dose combination formulation and harmonized weight bands.**

| **Weight Bands** | **150R, 75H, 400Z, 275E**† | **75R, 50H** |
| --- | --- | --- |
| 25.0 – 29.9 kg | 2 | 2 |
| 30.0 – 34.9 kg | 2 | 2 |

*Rifampicin (R):10mg/kg (range 8–12mg/kg) to a maximum dose 600mg/day; Isoniazid (H):5mg/kg (range 4–6mg/kg) to a maximum dose 300mg/day; Pyrazinamide (Z): 25mg/kg (20–30mg/kg); Ethambutol (E): range 15-20mg/kg) ; †Either HRZE or HR fixed dose combination with additional Z.

**Text C:** Harmonized weight band simulation results

Implementation of harmonized weight bands while using the current FDC will give drug exposures like current weight bands, with slight increase in exposure in children weighing 6-8, 10-12 and 14-16 kg.

**Figure C: Simulated drug exposures achieved after administration of WHO recommended doses with weight bands used in HIV.**


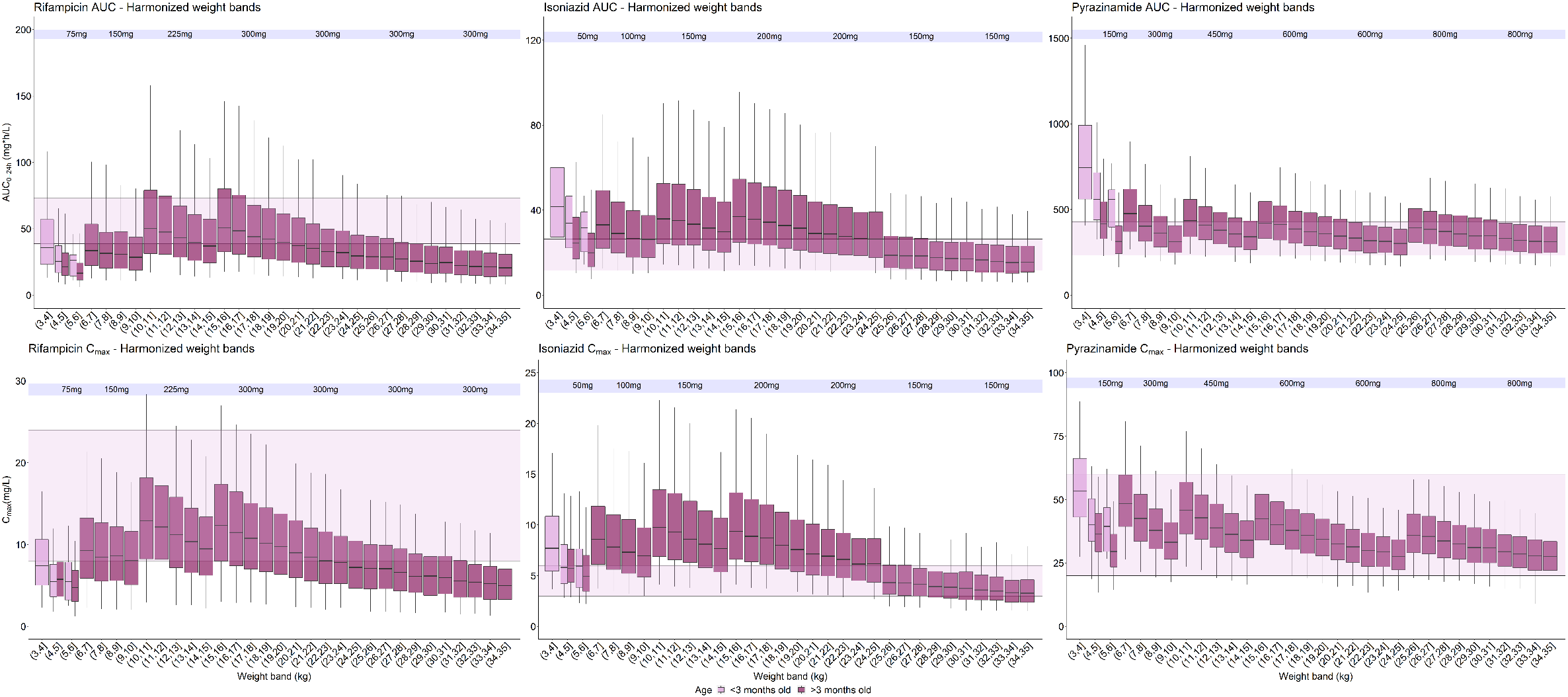


**Figure C:** Simulated rifampicin, isoniazid and pyrazinamide AUC from time zero to 24 h (AUC0–24) and maximum concentrations (Cmax) versus body weight, with concentrations achieved when dosed with World health organization (WHO) recommended doses and the existing fixed dose combination formulation, with weight bands used in HIV, after accounting for age and weight. The plum boxes represent patients <3 months while the maroon boxes represent patients ≥3 months. The plum horizontal shaded area represents the reported adult median AUC0-24 ranges (38.7-72.9, 11.6-26.3 and 233-429 mg*h/L) and Cmax ranges (8-24, 3-6, and 20-60 mg/L) for rifampicin, isoniazid and pyrazinamide respectively. The boxes indicate the inter-quartile range, while the whiskers denote the 2.5th and the 97.5th percentiles.

### Figure D: Visual Predictive Check of isoniazid concentration versus time after dose, stratified by HIV status and antiretroviral therapy.


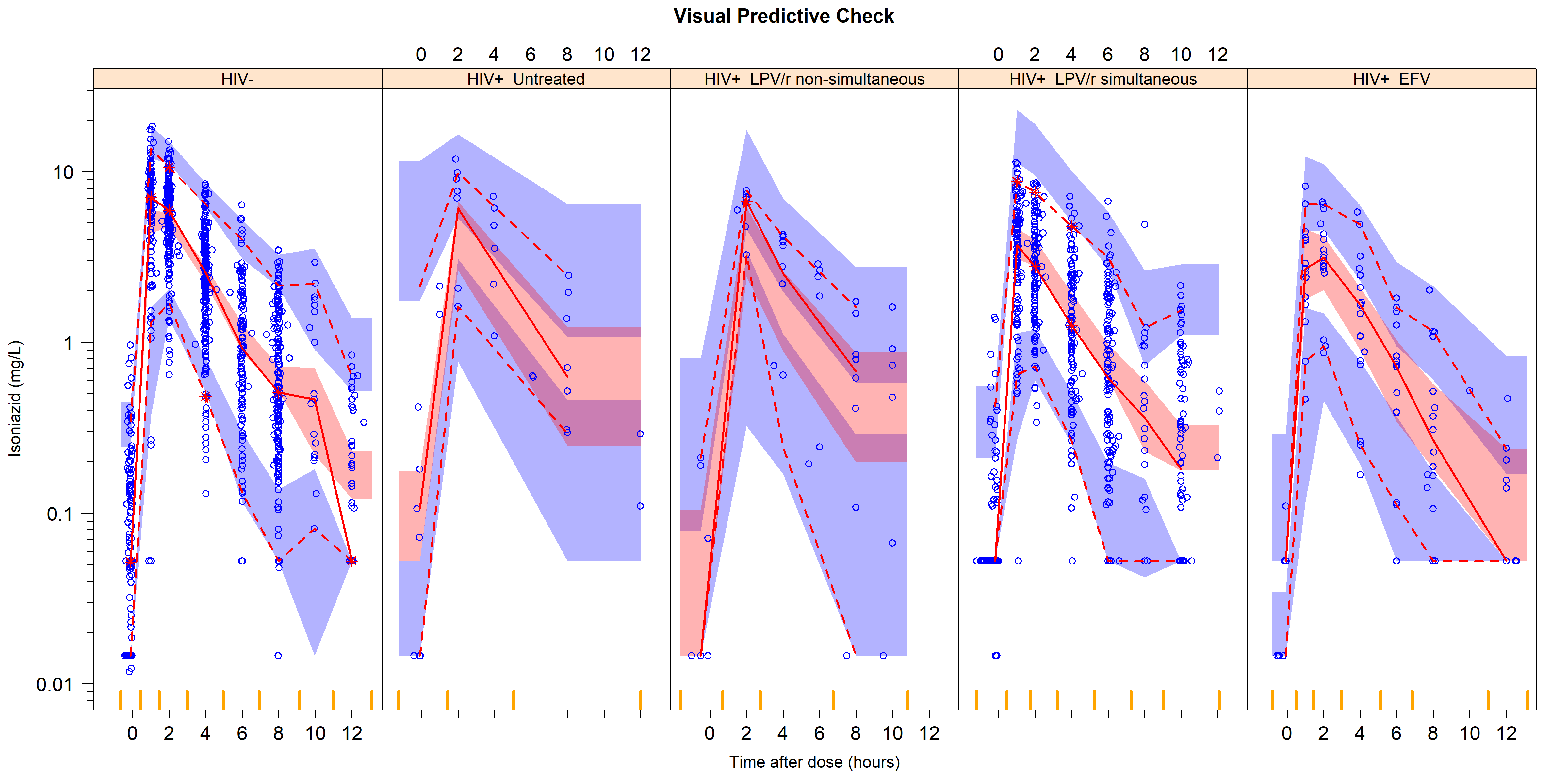


**Figure D:** The panels from left to right show the fit for children who were HIV-, HIV+ and not yet on antiretroviral therapy (ART), HIV+ on lopinavir/ritonavir that was taken at-least 2 hours before isoniazid, HIV+ on lopinavir/ritonavir taken at the same time with isoniazid and HIV+ and on efavirenz, after accounting for formulation, age, weight, study. The solid and dashed lines represent the 5th (red dashed), 50th (red solid), and 95th (red dashed) percentiles of the observed data, while the shaded areas represent the model-predicted 95% confidence intervals for the same percentiles (5th/95th blue shaded; 50th red shaded). The blue dots are the observed concentrations. LPV/r = lopinavir/ritonavir. EFV = efavirenz.

**Figure E: Visual Predictive Check of drug concentration versus time after dose, stratified by study.**


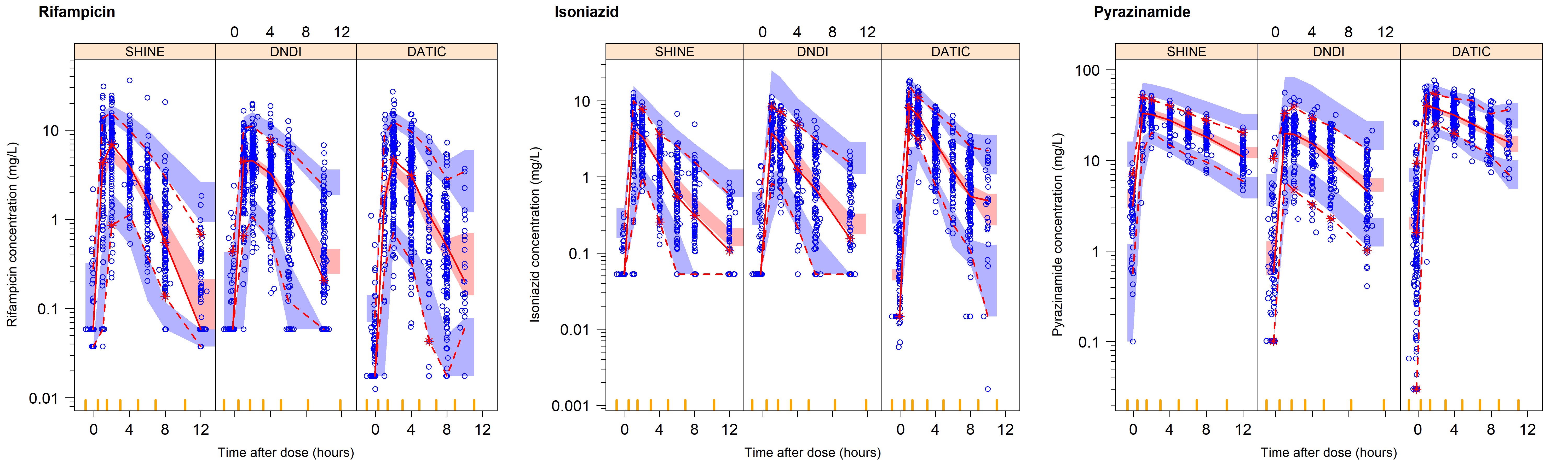


**Figure E**: The plots show the final model fit for rifampicin, isoniazid, and pyrazinamide respectively. The solid and dashed lines represent the 5th (red dashed), 50th (red solid), and 95th (red dashed) percentiles of the observed data, while the shaded areas represent the model-predicted 95% confidence intervals for the same percentiles (5th/95th blue shaded; 50th red shaded). The blue dots are the observed concentrations.

### Figure F: Visual Predictive Check of rifampicin drug concentration versus time after dose, stratified by lopinavir/ritonavir.


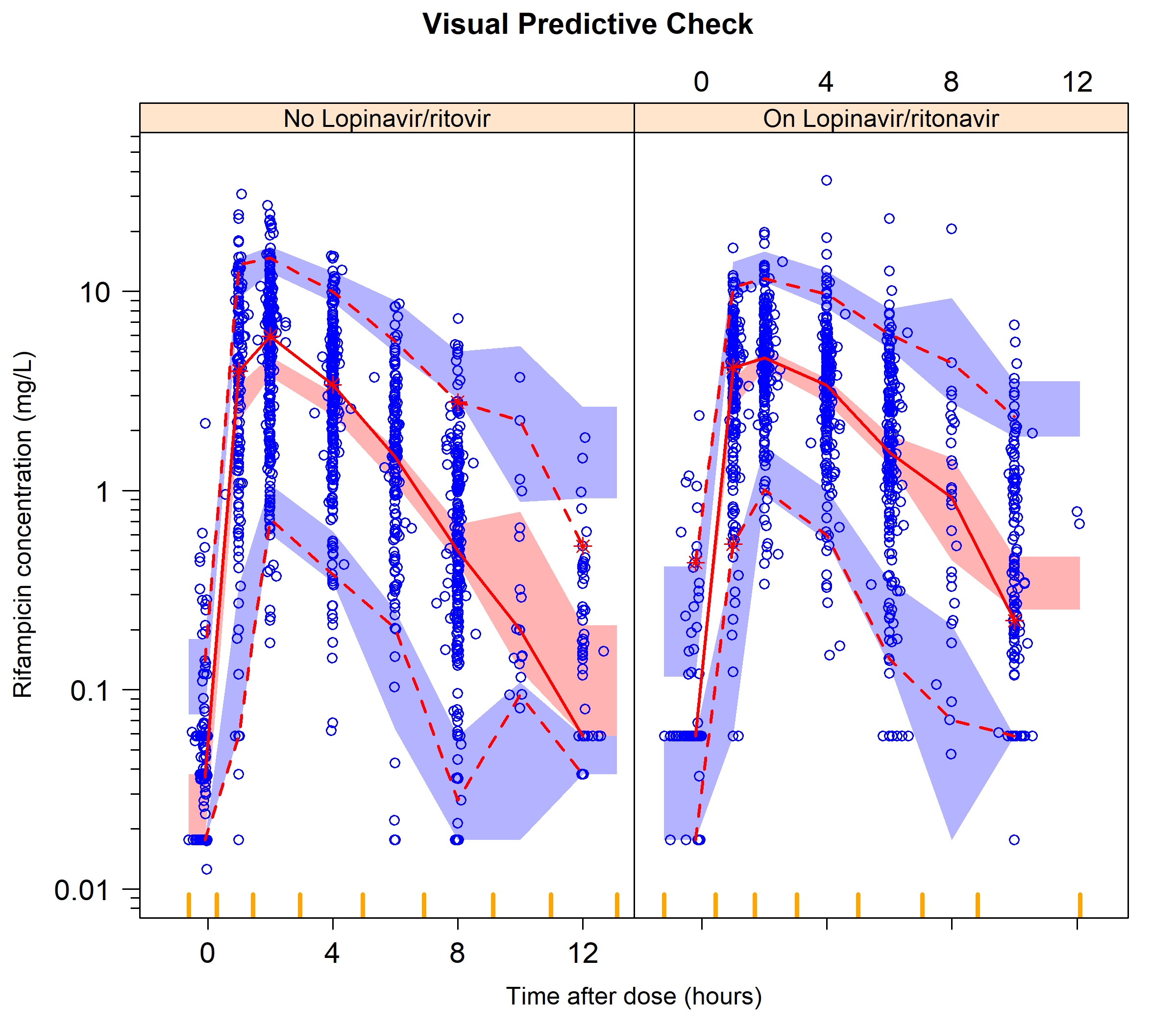


**Figure F:** The plots on the left and right show the final model fit for children not on lopinavir/ritonavir and those on lopinavir/ritonavir, respectively, after accounting for formulation, age, weight, and laboratory. The solid and dashed lines represent the 5th (red dashed), 50th (red solid), and 95th (red dashed) percentiles of the observed data, while the shaded areas represent the model-predicted 95% confidence intervals for the same percentiles (5th/95th blue shaded; 50th red shaded). The blue dots are the observed concentrations.

### Figure G: Visual Predictive Check of pyrazinamide drug concentration versus time after dose, stratified by lopinavir/ritonavir.


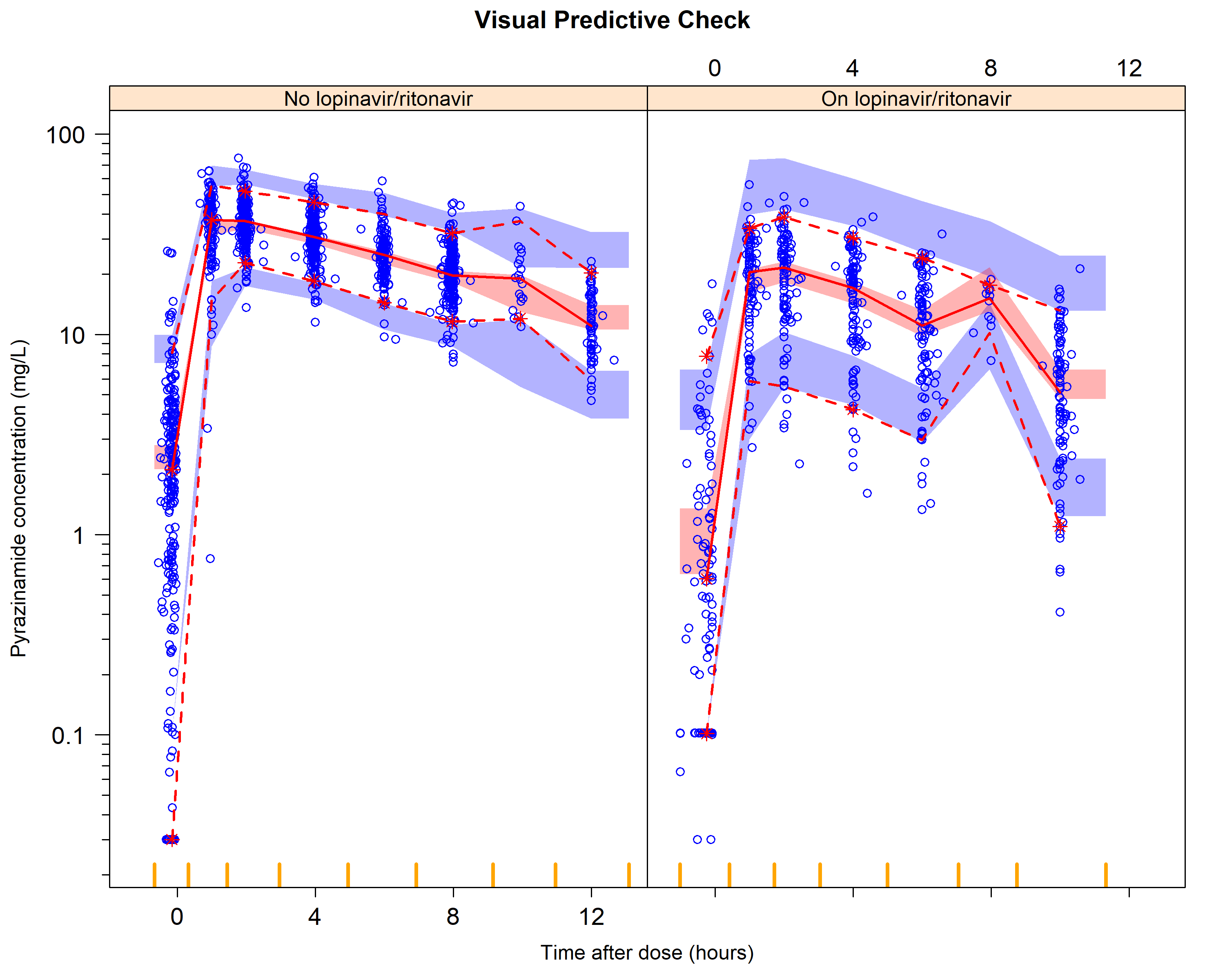


**Figure G:** The plots on the left and right show the final model fit for children not on lopinavir/ritonavir and those on lopinavir/ritonavir, respectively, after accounting for mode of drug administration (crushing tablets), age, fat-free mass, study, and laboratory. The solid and dashed lines represent the 5th (red dashed), 50th (red solid), and 95th (red dashed) percentiles of the observed data, while the shaded areas represent the model-predicted 95% confidence intervals for the same percentiles (5th/95th blue shaded; 50th red shaded). The blue dots are the observed concentrations. The visual artefact of the appearance of an increasing concentration around 10 hours is caused by a sub-group of younger children who had a sample taken at 10 hours.

# **Text D: Genotyping**

# NAT2 genotype information was available only for the DATiC study [2], where genotyping was performed in consenting children recruited at the Desmond Tutu TB Centre (DTTC) and Red Cross Children’s Hospital (RedX). In children from DTTC, only N-acetyltransferase 2 (NAT2) genotype was evaluated according to a previously described method for the NAT2*5, NAT2*6, NAT2*7, NAT2*12, NAT2*13, and NAT2*14 alleles [3]. Children from RedX were genotyped for one single-nucleotide polymorphism (SNP) for NAT2, Arylacetamide deacetylase and the SLCO1B1 gene thusly; Genomic DNA was extracted from frozen samples of whole blood using a commercial kit (QIAamp DNA Blood Maxi, Qiagen Inc., Hilden, Germany). Extracted DNA was stored at -70° C prior to further analysis. DNA sample purity was assessed using the A260/A280 ratio and normalized to 20 ng/mL prior to PCR. Real time PCR was performed for the following single nucleotide polymorphisms using commercial kits (Applied Biosystems Inc. Warrington, United Kingdom): tag SNP located 14 kb 3’ of the NAT2 exon (rs1495741), SLCO1B1 (rs4149032) and AADAC (rs1803155) [4-7]. PCR amplification was performed in an Opticon 2.0 thermal cycler (Bio-Rad Laboratories, Inc., United Kingdom). Genotypes were analysed using MJ Opticon Monitor analysis software (version 3.1.32, Bio-Rad Laboratories, Inc., United Kingdom) using the C(t) values for the dyes FAM and VIC corresponding to the different alleles to obtain allelic discrimination plots and make allele calls.

**References**

1. World Health Organization (WHO). Rapid advice : treatment of tuberculosis in children. 2010; [accessed 2021 May 17]. Available from: https://apps.who.int/iris/handle/10665/44444

2. Denti P, Wasmann RE, van Rie A, Winckler J, Bekker A, Rabie H, et al. Optimizing Dosing and Fixed-Dose Combinations of Rifampicin, Isoniazid, and Pyrazinamide in Pediatric Patients With Tuberculosis: A Prospective Population Pharmacokinetic Study. Clin Infect Dis. 2022 Aug 24;75(1):141–51. doi: ﻿10.1093/cid/ciab908

3. Bekker A, Schaaf HS, Seifart HI, Draper HR, Werely CJ, Cotton MF, et al. Pharmacokinetics of Isoniazid in Low-Birth-Weight and Premature Infants. Antimicrob Agents Chemother. 2014 Apr;58(4):2229–34. doi: ﻿10.1128/AAC.01532-13

4. Sabbagh A, Darlu P, Vidaud M. Evaluating NAT2PRED for inferring the individual acetylation status from unphased genotype data. BMC Med Genet. 2009 Dec 31;10(1):148. doi: ﻿10.1186/1471-2350-10-148

5. García-Closas M, Hein DW, Silverman D, Malats N, Yeager M, Jacobs K, et al. A single nucleotide polymorphism tags variation in the arylamine N-acetyltransferase 2 phenotype in populations of European background. Pharmacogenet Genomics. 2011 Apr;21(4):231–6. doi: ﻿10.1097/FPC.0b013e32833e1b54

6. Sloan DJ, McCallum AD, Schipani A, Egan D, Mwandumba HC, Ward SA, et al. Genetic Determinants of the Pharmacokinetic Variability of Rifampin in Malawian Adults with Pulmonary Tuberculosis. Antimicrob Agents Chemother. 2017 Jul;61(7):1–9. doi: ﻿10.1128/AAC.00210-17

7. Ho H-T, Wang T-H, Hsiong C-H, Perng W-C, Wang N-C, Huang T-Y, et al. The NAT2 tag SNP rs1495741 correlates with the susceptibility of antituberculosis drug-induced hepatotoxicity. Pharmacogenet Genomics. 2013 Apr;23(4):200–7. doi: ﻿10.1097/FPC.0b013e32835e95e1

# **Text E: Modeling code**

## **Rifampicin model**

;; 1. Based on: run000

;; 2. Description: Rifampicin Model

$PROBLEM PAED_RIF

$INPUT ID VISIT DAT2=DROP TIME EVID OCCASSION DV MDV BLQ CENS AMT AGE

WEIGHT LAB FORMULATION STUDY FFM INITIALIZED LOPINAVIR_RITONAVIR

$DATA DATA.csv IGNORE=@

$SUBROUTINE ADVAN15 TRANS1

TOL=9

ATOL=9

$MODEL

NCOMPARTMENTS=3

COMP=(ABS DEFDOSE)

COMP=(LIVER)

COMP=(CENTRAL DEFOBSERVATION)

;PRIOR--------------------------------------------------------------------

;Sim_start

$PRIOR NWPRI NPEXP=1 PLEV=0.9999

;Sim_end

$PK

;------------------------BSV------------------------------------------------

BSVCL = ETA(1)

BSVV = ETA(2)

BSVBIO = ETA(3)

BSVKA = ETA(4)

BSVMTT = ETA(5)

BSVNN = ETA(6)

BSVQH = ETA(7)

BSVFU = ETA(8)

BSVVH = ETA(9)

BSVKM = ETA(10)

;------------------------BOV----------------------------------------------

BOVBIO = 0

BOVKA = 0

BOVMTT = 0

IF(OCC.EQ.1) BOVBIO = ETA(11)

IF(OCC.EQ.2) BOVBIO = ETA(12)

IF(OCC.EQ.3) BOVBIO = ETA(13)

IF(OCC.EQ.4) BOVBIO = ETA(14)

IF(OCC.EQ.1) BOVKA = ETA(15)

IF(OCC.EQ.2) BOVKA = ETA(16)

IF(OCC.EQ.3) BOVKA = ETA(17)

IF(OCC.EQ.4) BOVKA = ETA(18)

IF(OCC.EQ.1) BOVMTT = ETA(19)

IF(OCC.EQ.2) BOVMTT = ETA(20)

IF(OCC.EQ.3) BOVMTT = ETA(21)

IF(OCC.EQ.4) BOVMTT = ETA(22)

;------- Typical values of covariates---------------------------------------

TVWT = 14.0 ;Typical value of weight

TVFFM = 10.7 ;Typical value of fat free mass

;--------- Allometric scaling and covariates--------------------------------

ALLMCL_WT = (WT/TVWT)**0.75 ;For clearance

ALLMV_WT = (WT/TVWT) ;For volume

;----------Allometry for liver----------------------------------------------

ALLMCL_WT_HEP = (WT/70)**0.75 ;For clearance

ALLMV_WT_HEP = (WT/70) ;For volume

;------- Maturation of clearance--------------------------------------------

AGEM = AGE*12 ;to convert age to months

PGA = AGEM + 9 ;to add 9 months

LOGPGA50 = THETA(1)

GAMMA = THETA(2)

MATCL = 0

IF (PGA>0) MATCL=1/(1+EXP(-EXP(GAMMA)*(LOG(PGA)-LOGPGA50)))

;--------------------Age on Bio----------------------------------------------

AGE_BIO_BR = THETA(3) ;Bio at birth

BIO_BIRTH = THETA(4) ;Breakpoint

AGE_BIO_SL = (1-BIO_BIRTH) / AGE_BIO_BR

AGE_BIO = 1

IF (AGEM<AGE_BIO_BR) AGE_BIO = BIO_BIRTH + AGE_BIO_SL * AGE_ADJ_GEST

;--------------LOPINAVIR_RITONAVIR on CL----------------------------

LPV_CL = 1

IF (LOPINAVIR_RITONAVIR.EQ.1) LPV_CL=THETA(14) ;On LOPINAVIR_RITONAVIR

L_LPV_CL = LOG(LPV_CL)

;-------------------------------Formulation on BIO---------------------------

FORM_BIO = 1

IF(FORMULATION.EQ.1)FORM_BIO = THETA(15) ; DATIC R_CIN

L_FORM_BIO = LOG(FORM_BIO)

;---------MU Definitions----------------------------------------------------

MU_1 = THETA(5)+L_LPV_CL ; CLEARANCE

MU_2 = THETA(6) ; CENTRAL VOL

MU_3 = THETA(7)+L_FORM_BIO ; BIOAVAILABILITY

MU_4 = THETA(8) ; ABS. RATE CONSTANT

MU_5 = THETA(11) ; MTT

MU_6 = THETA(12) ; NUMBER OF TRANSIT COMPARTMENTS

;----------------MU Definitions for hepatic parameters-------------------

MU_7 = THETA(16) ; PLASMA FLOW RATE

MU_8 = THETA(17) ; UNBOUND PLASMA FRACTION OF RIF

MU_9 = THETA(18) ; LIVER VOLUME

MU_10 = THETA(19) ; LOG KM

;-----------Define parameters------------------------------------------------

CL = EXP(MU_1 + ETA(1))*MATCL*ALLMCL_WT ; CLEARANCE

V = EXP(MU_2 + ETA(2))*ALLMV_WT ; CENTRAL VOL.

BIO = EXP(MU_3 + ETA(3))*EXP(BOVBIO)*AGE_BIO ; BIOAVAILABILITY

KA = EXP(MU_4 + ETA(4))*EXP(BOVKA) ; ABS. RATE CONSTANT

MTT = EXP(MU_5 + ETA(5))*EXP(BOVMTT) ; MTT TIME

NN = EXP(MU_6 + ETA(6)) ; Number of transit compartments

QH = EXP(MU_7 + ETA(7))*ALLMCL_WT_HEP ;INTERCOMPARTMENTAL CLEARANCE

FU = EXP(MU_8 + ETA(8)) ; FRACTION UNBOUND

VH = EXP(MU_9 + ETA(9))*ALLMV_WT_HEP ; HEPATIC VOLUME

KM = EXP(MU_10 + ETA(10)); MICHAELIS MENTEN RATE CONSTANT

CLINT = CL ; INTRINSIC CLEARANCE

VMAX = CLINT*KM ; MAX ENZYMATIC RATE FROM EQ. CLint = Vmax/KM

;--------------------------Transit compartment absorption--------------------

F1=0 ; I need to set bioavailability in compartment 1 to 0 for this implementation of the transit compartment absorption

KTR = (NN+1)/MTT ; The number of actual transit compartments is NN+1, so this number can never be 0

IF (NEWIND/=2.OR.EVID>=3) THEN ; new individual, or reset event

; The values read here will be stored in TDOS and PD in this very PK call.

TNXD=TIME ; Time of the dose

PNXD=AMT ; Amount. If it's zero, the DE is deactivated.

ENDIF

TDOS=TNXD ; This will either save here the temporary values if it's a new individual...

PD=PNXD ; ...or the values which were read one record ahead during the execution of the previous record.

IF(AMT>0) THEN ; This reads one record ahead and stores the data to be used when running the following record

TNXD=TIME

PNXD=AMT

ENDIF

; To speed up the computation, I calculate here all the non-time-varying quantities used in $DES

PIZZA = LOG(BIO*PD*KTR + 0.00001) - GAMLN(NN+1) ; without +0.00001, it won't work with ETAs in bioavailability

$DES

CP = A(3)/V ; drug concentration in Plasma

TEMPO = T-TDOS ; this is time after dose for the transit, it should always be >= 0

KTT = 0

TRANSIT = 0

IF(PD.GT.0.AND.TEMPO.GT.0) THEN ; This happens only id PD>0, so only if a dose has been detected

KTT = KTR*(TEMPO)

TRANSIT = EXP(PIZZA+NN*LOG(KTT)-KTT)

ENDIF

;-------------------Saturable liver -----------------------------------------

CH = A(2)/VH ; drug concentration in liver

; Set saturable elimination to zero for patients with negative concentrations in liver

; product of a logarithm cannot be negative - this would not work

; normal Michalis-Menten equation gives you a rate =k*A = CL*C

; but we want to get CL, so you divide the rate by concentration - and get saturable clearance

; equation is placed in $DES because saturable clearance changes over time

SAT_CL = 0

IF (CH>0) SAT_CL = VMAX / (CH + KM)

EH = (SAT_CL*FU)/((SAT_CL*FU)+QH) ; fraction undergoing first pass extraction

FH = 1 - EH ;fraction available after 1st pass to go to systemic circulation

K20=(QH*EH/VH) ;rate of elimination from the liver compartment

K23= (QH*FH/VH) ;rate of transfer from liver to central compartment

K32=(QH/V) ;rate of transfer from central compartment to the liver compartment

DADT(1) = TRANSIT -KA*A(1) ;change of drug amount in absorption compartment over time

DADT(2) = KA*A(1) -K20*A(2) -K23*A(2) +K32*A(3) ;change of drug amount in liver compartment over time

DADT(3) = K23*A(2) -K32*A(3) ;change of drug amount in central compartment over time

$ERROR

IPRED=A(3)/V

;UCT lab

LLOQ = 0.117 ; DEFINE YOUR OWN LLOQ HERE

CENS_THR = LLOQ

;DATIC

IF (STUDY.EQ.3) THEN

CENS_THR = 0.3*LLOQ ; inferred LOD = 30% of LLOQ since LOD not provided.

ENDIF

;India lab

IF (LAB.EQ.2) THEN

IPRED = (A(3)/V)*THETA(13)

LLOQ = 0.250 ; DEFINE YOUR OWN LLOQ HERE

CENS_THR = 0.3*LLOQ ; inferred LOD = 30% of LLOQ since LOD not provided.

ENDIF

PROP = IPRED*THETA(9) ;proportional error

ADD = THETA(10)+(LLOQ*0.2) ;additive error

IF (ICALL/=4.AND.CENS==1) THEN

ADD = ADD+(CENS_THR*0.5)

ENDIF

NO_FIT=0

; CENS==1 are the BLQ samples kept in the fit. When you have a serires, keep the last one before the cmax and the first one after the cmax

; CENS==2 are the remaining BLQ samples (Below LOD) in series, which I want to disergard, but yet include for diagnostic purposes

IF (ICALL/=4.AND.CENS==2) THEN

PROP = 0

ADD = 10000000000

NO_FIT=1

; Using this large error has the same effect as ignoring, expect the record is still there, so I can use it in VPCs

ENDIF

W = SQRT(ADD**2+PROP**2)

; Protective code

IF (W.LE.0.000001) W=0.000001

IRES=DV-IPRED

IWRES=IRES/W

Y = IPRED + W*ERR(1)

; For simulation, like in case of VPC

IF (ICALL==4.AND.Y<=CENS_THR) THEN

Y = CENS_THR/2

ENDIF

; To calculate time after dose

IF(AMT>0) THEN

TIMEDOSE = TIME

AMOUNTDOSE = AMT

ENDIF

$THETA

2.3969 ; 1 LOGPGA50 months [log]

1.18484 ; 2 GAMMA [log]

3.32132 ; 3 AGE_BIO_BR

0.48919 ; 4 BIO BIR

3.95571 ; 5 CL [L/h] [log]

2.62098 ; 6 V [L] [log]

0 FIX ; 7 BIO [log]

1.00456 ; 8 KA [1/h] [log]

0.241248 ; 9 PROP []

0 FIX ; 10 ADD [mg/L]

-0.663645 ; 11 MTT [log]

2.9205 ; 12 NN [log]

1.1979 ; 13 Lab scaling India Vs UCT

0.796128 ; 14 LPV_CL

0.367488 ; 15 R_CIN BIO

4.49980967 FIX ; 16 QH [log]

-1.60943791 FIX ; 17 FU [log]

0 FIX ; 18 VH [log]

2.2386 ; 19 KM [log]

; PRIORS---------------------------

;Sim_start

$THETAP

2.48 FIX ; 1 PGA50 months [log]

1.27 FIX ; 2 GAMMA [log]

;Sim_end

;---------------------------------------

; UNCERTAINTY IN PRIORS

;Sim_start

$THETAPV BLOCK(2) FIX

0.01 ; LOGPGA50

0 0.01 ; GAMMA

;Sim_end

;------------------------------------------------------------------------------------------------------------------------------------------------------

$OMEGA BLOCK(1)

0.122149 ; 1 BSV CL

$OMEGA BLOCK(1) FIX

0.0001 ; 2 BSV V

$OMEGA BLOCK(1) FIX

0.0001 ; 3 BSV BIO

$OMEGA BLOCK(1) FIX

0.0001 ; 4 BSV KA

$OMEGA BLOCK(1) FIX

0.0001 ; 5 BSV MTT

$OMEGA BLOCK(1) FIX

0.0001 ; 6 BSV NN

$OMEGA BLOCK(1) FIX

0.0001 ; 7 BSV QH

$OMEGA BLOCK(1) FIX

0.0001 ; 8 BSV FU

$OMEGA BLOCK(1) FIX

0.0001 ; 9 BSV VH

$OMEGA BLOCK(1) FIX

0.0001 ; 10 BSV KM

$OMEGA BLOCK(1)

0.124656 ; 11 BOVBIO

$OMEGA BLOCK(1) SAME

$OMEGA BLOCK(1) SAME

$OMEGA BLOCK(1) SAME

;-------------------------------------------------------------------------------------------------------------------------------------------------------

$OMEGA BLOCK(1)

2.62634 ; 15 BOVKA

$OMEGA BLOCK(1) SAME

$OMEGA BLOCK(1) SAME

$OMEGA BLOCK(1) SAME

;-------------------------------------------------------------------------------------------------------------------------------------------------------

$OMEGA BLOCK(1)

0.177146 ; 19 BOVMTT

$OMEGA BLOCK(1) SAME

$OMEGA BLOCK(1) SAME

$OMEGA BLOCK(1) SAME

;--------------------------------------------------------------------------------------------------------------------------------------------------------

$SIGMA 1 FIX

;-------------------------------------------------------------------------------------------------------------------------------------------------------

$ESTIMATION METHOD=SAEM GRD=TS(9,10) SIGL=5 PRINT=1

NITER=5000 NOPRIOR=0 NBURN=5000 CTYPE=3

MUM=M(5-8,11-12,16-19):N(9,10):D(1-4,13-15)

$ESTIMATION METHOD=IMP GRD=TS(9,10) SIGL=5 EONLY=2 ISAMPLE=10000

MUM=M(5-8,11-12,16-19):N(9,10):D(1-4,13-15) NOPRIOR=0

PRINT=1 NITER=500 MAPITER=0 CTYPE=3

## **Isoniazid model**

;; 1. Based on: run000

;; 2. Description: INH model

$PROBLEM PAED_INH

$INPUT ID DAT2=DROP TIME EVID OCCASSION DV MDV BLQ CENS AMT AGE WEIGHT

LAB FORMULATION STUDY FFM LOPINAVIR_RITONAVIR EFAVIRENZ

$DATA DATA.csv IGNORE=@

$SUBROUTINE ADVAN5 TRANS1

$MODEL NCOMPARTMENTS=14

COMP=(TRANSIT1,DEFDOSE) ;1 GUT TRANIST 1 (F1 associated with first comp)

COMP=(TRANSIT2) ;2 GUT TRANIST 2

COMP=(TRANSIT3) ;3 GUT TRANIST 3

COMP=(TRANSIT4) ;4 GUT TRANIST 4

COMP=(TRANSIT5) ;5 GUT TRANIST 5

COMP=(TRANSIT6) ;6 GUT TRANIST 6

COMP=(TRANSIT7) ;7 GUT TRANIST 7

COMP=(TRANSIT8) ;8 GUT TRANIST 8

COMP=(TRANSIT9) ;9 GUT TRANIST 9

COMP=(TRANSIT10) ;10 GUT TRANIST 10

COMP=(TRANSIT11) ;11 GUT TRANIST 11

COMP=(ABS) ;12 GUT ABS

COMP=("CENTRAL",DEFOBS) ;13 CENTRAL CMT

COMP=(PERI1) ;14 PERIPHERAL CMT

;--------------PRIOR---------------------------------------------------------------------------

;Sim_start

$PRIOR NWPRI NPEXP=1 PLEV=0.9999

;Sim_end

$MIX

NSPOP=3 ;3 Subpopulations

P(1) = THETA(14) ; Probability of being in fast acetylator subpupulation

P(2) = THETA(19) ; Probability of being in intermediate acetylator subpupulation

P(3) = 1 - THETA(14)-THETA(19) ; Probability of being in slow acetylator subpupulation

;-------------------------------------------------------------------------------------------------

$PK

; ------- BETWEEN SUBJECT VARIABILITY(BSV)

BSVCL = ETA(1)

BSVV = ETA(2)

; ---------- BETWEEN OCCASSION VARIABILITY(BOV)

BOVBIO = 0

BOVKA = 0

BOVMTT = 0

IF(OCCASSION.EQ.1) BOVBIO = ETA(3)

IF(OCCASSION.EQ.2) BOVBIO = ETA(4)

IF(OCCASSION.EQ.3) BOVBIO = ETA(5)

IF(OCCASSION.EQ.4) BOVBIO = ETA(6)

IF(OCCASSION.EQ1) BOVKA = ETA(7)

IF(OCCASSION.EQ.2) BOVKA = ETA(8)

IF(OCCASSION.EQ.3) BOVKA = ETA(9)

IF(OCCASSION.EQ.4) BOVKA = ETA(10)

IF(OCCASSION.EQ.1) BOVMTT = ETA(11)

IF(OCCASSION.EQ.2) BOVMTT = ETA(12)

IF(OCCASSION.EQ.3) BOVMTT = ETA(13)

IF(OCCASSION.EQ.4) BOVMTT = ETA(14)

;--------- Allometric scaling and covariates

; ------- Typical values of covariates

TVWT = 14 ;Weight

TVFFM = 10 ;Fat free mass

;--------- Allometric scaling

ALLMCL_WT = (WT/TVWT)**0.75

ALLMV_WT = (WT/TVWT)

ALLMCL_FFM = (FFM/TVFFM)**0.75

ALLMV_FFM = (FFM/TVFFM)

;------- Maturation of clearance

PGA = AGE + 9 ; to add 9 months

LOGPGA50 = THETA(1)

GAMMA = THETA(2)

MATCL = 0

IF (PGA>0) MATCL=1/(1+EXP(-EXP(GAMMA)*(LOG(PGA)-LOGPGA50)))

;------------------------------Mixture Modeling-------------------------------------------------------

NAT2 = NAT2_PRED ;0 Fast, 1 Intermediate, 2 Slow, -1 Missing

EST = MIXEST

IMP_NAT = 0 ;IMPUTED_Fast

IF(MIXNUM.EQ.2) IMP_NAT = 1 ;IMPUTED_Intermediate

IF(MIXNUM.EQ.3) IMP_NAT = 2 ;IMPUTED_slow

IF(NAT2.LT.0)NAT2_MIX= IMP_NAT ;-1 = Missing genotype information therefore use the imputed

IF(NAT2.GE.0)NAT2_MIX= NAT2 ;With genotype information

IF(NAT2_MIX.EQ.0) THEN

TVCL = THETA(3)*ALLMCL_WT*MATCL ; fast

ENDIF

IF (NAT2_MIX.EQ.1) THEN

TVCL = THETA(13)*ALLMCL_WT*MATCL ; Intermediate

ENDIF

IF (NAT2_MIX.EQ.2) THEN

TVCL = THETA(20)*ALLMCL_WT*MATCL ; slow

ENDIF

;-------------------------------efavirenz on BIO--------------------------------------------------

EFV_BIO = 1

IF(EFAVIRENZ.EQ.1)EFV_BIO = THETA(15) ;On efavirenz

;-------------------------------FORMULATION ON BIO-------------------------------------------------

FORM_BIO = 1

IF(FORMULATION.EQ.1)FORM_BIO = THETA(16) ;RHZE or RH_McLeaod's formulation

;--------------------AGE ON BIO---------------------------------------------------------------------

BIO_BIRTH = THETA(17);Bio at birth

AGE_BIO_BR = THETA(18);Breakpoint

AGE_BIO_SL = (1-BIO_BIRTH) / AGE_BIO_BR

AGE_BIO = 1

IF (AGEM<AGE_BIO_BR) AGE_BIO = BIO_BIRTH + AGE_BIO_SL * AGEM

;--------------Scaling BOV_BIO-----------------------------------------------------------------------

BOVBIO_DN = BOVBIO

IF(STUDY.EQ.2) BOVBIO_DN = BOVBIO*THETA(21) ;DNDI_BOVBIO

;-----------LOPINAVIR_RITONAVIR ON BIO--------------------------------------------------------------

LPV_BIO = 1

IF(LOPINAVIR_RITONAVIR.EQ.1)LPV_BIO = THETA(22) ;On Lopinavir/ritonavir

;---------Typical values-----------------------------------------------------------------------------

TVV = THETA(4)*ALLMV_WT

TVKA = THETA(5)

TVBIO = THETA(6)*EFV_BIO*FORM_BIO*AGE_BIO*LPV_BIO

TVMTT = THETA(9)

TVV3 = THETA(10)*ALLMV_WT

TVQ = THETA(11)*ALLMCL_WT

TVNN = THETA(12)

;-----------Define parameters------------------------------------------------------------------------

CL = TVCL*EXP(BSVCL) ; CLEARANCE

V = TVV*EXP(BSVV) ; CENTRAL VOLUME

KA = TVKA*EXP(BOVKA) ; ABSORPTION RATE CONSTANT

BIO = TVBIO*EXP(BOVBIO) ; BIOAVAILABILITY

MTT = TVMTT*EXP(BOVMTT) ; MTT TIME

V3 = TVV3 ; PERIPH VOLUME

Q = TVQ ; INTER COMPARTMENTAL CLEARANCE

NN = TVNN ; Number of transit compartments

;------------Rate constants---------------------------------------------------------------------------

F1 = BIO

KTR = (NN+1)/MTT

K12 = KTR ;Rate between transit CMT

K23 = KTR ;Rate between transit CMT

K34 = KTR ;Rate between transit CMT

K45 = KTR ;Rate between transit CMT

K56 = KTR ;Rate between transit CMT

K67 = KTR ;Rate between transit CMT

K78 = KTR ;Rate between transit CMT

K89 = KTR ;Rate between transit CMT

K910 = KTR ;Rate between transit CMT

K1011 = KTR ;Rate between transit CMT

K1112 = KTR ;Rate between transit CMT

K1213 = KA ;Absorption rate constant

K130 = CL/V ;Elimination rate constant

K1314 = Q/V ;(rate constant from central to peripheral 1)

K1413 = Q/V3 ;(rate constant from peripheral 1 to central)

S13 = V ;CENETRAL COMPARTMENT SCALAR (based on numbering in $MODEL)

;----------------------------------------------------------------------------------------------------

$ERROR

IPRED=A(13)/V

LLOQ = 0.105

CENS_THR = LLOQ

IF (STUDY.EQ.3) THEN ;DATiC

LLOQ = 0.0977

CENS_THR = 0.02931 ; inferred INH LOD = 30% of LLOQ

ENDIF

PROP = IPRED*THETA(7)

ADD = THETA(8)+(LLOQ*0.2)

; For CENS==1 (i.e. first CENSORED value in a series, which was imputed to CENS_THR/2), we add extra ;additive error on the concentrations, since the value in DV has been imputed and therefore more ;uncertain.

IF (ICALL/=4.AND.CENS==1) THEN

ADD = ADD +(CENS_THR*0.5)

ENDIF

NO_FIT = 0

; For CENS==2 (i.e. the trailing CENSORED values in a series that were imputed to CENS_THR/2), we ;don't want these to influence the fit, we only want them for simulation-based diagnostics.

IF (ICALL/=4.AND.CENS==2) THEN

PROP = 0

ADD = 10000000000

NO_FIT = 1

; Using this large error has the same effect as ignoring, expect the record is still there, so I can ;use it in VPCs

ENDIF

W = SQRT(ADD**2+PROP**2)

; Protective code

IF (W.LE.0.000001) W=0.000001

IRES=DV-IPRED

IWRES=IRES/W

Y = IPRED + W*ERR(1)

; To prevent simulation (ICALL==4) of negative values. It set a positive lower bound for Y, so that ;VPCs in the log-scale can be plotted

IF (ICALL==4.AND.Y<=CENS_THR)

Y = CENS_THR/2

ENDIF

;--------------------------------------------------------------------------------------------------

$THETA

2.3885 ; 1 PGA50 months [log]

1.19336 ; 2 GAMMA [log]

7.06086 ; 3 CL_FAST [L/h]

13.8101 ; 4 V [L]

3.56262 ; 5 KA [1/h]

1 FIX ; 6 BIO

0.142232 ; 7 PROP []

0 FIX ; 8 ADD [mg/L]

0.300294 ; 9 MTT

5.72896 ; 10 V3 [L]

0.508755 ; 11 Q [L/h]

10 FIX ; 12 NN []

6.15631 ; 13 CL_INTERMEDIATE [L/h]

0.14 FIX ; 14 Prob_fast

0.604936 ; 15 EFV_BIO

0.626442 ; 16 BIO_McLeaod's_Adult_formulation

0.647307 ; 17 BIO_BIR

26.4461 ; 18 AGE_BIO BR

0.42 FIX ; 19 Prob_INTR

4.03074 ; 20 CL_SLOW [L/h]

1.76164 ; 21 DNDI_BOVBIO

0.609381 ; 22 LPV_BIO

; PRIORS

;Sim_start

$THETAP

2.48 FIX ; 1 PGA50 months [log]

1.27 FIX ; 2 GAMMA [log]

;Sim_end

;----------------------------------------------------------------------------------------------------

; UNCERTAINTY IN PRIORS

;Sim_start

$THETAPV BLOCK(2) FIX

0.01 ; PGA50

0 0.01 ; GAMMA

;Sim_end

;-----------------------------------------------------------------------------------------------------

$OMEGA BLOCK(1)

0.0744763 ; 1 BSVCL

$OMEGA BLOCK(1)

0.046334 ; 2 BSVV

;-----------------------------------------------------------------------------------------------------

$OMEGA BLOCK(1)

0.201188 ; 3 BOVBIO

$OMEGA BLOCK(1) SAME

$OMEGA BLOCK(1) SAME

$OMEGA BLOCK(1) SAME

;-----------------------------------------------------------------------------------------------------

$OMEGA BLOCK(1)

0.672536 ; 7 BOVKA

$OMEGA BLOCK(1) SAME

$OMEGA BLOCK(1) SAME

$OMEGA BLOCK(1) SAME

;-----------------------------------------------------------------------------------------------------

$OMEGA BLOCK(1)

0.66197 ; 11 BOVMTT

$OMEGA BLOCK(1) SAME

$OMEGA BLOCK(1) SAME

$OMEGA BLOCK(1) SAME

;-----------------------------------------------------------------------------------------------------

$SIGMA 1 FIX

;-----------------------------------------------------------------------------------------------------

$ESTIMATION MSFO=run001.msf MAXEVAL=9999 PRINT=1 METHOD=1 INTER NOABORT NSIG=3 SIGL=9 NONINFETA=1 ETASTYPE=1

## **Pyrazinamide model**

;; 1. Based on: 000

;; 2. Description: Pyrazinamide model

$PROBLEM PAED_PZA

$INPUT ID DAT2=DROP TIME EVID OCCASSION DV MDV BLQ CENS AMT AGE WEIGHT

LAB METHOD_OF_DRUG_ADMIN STUDY FFM LOPINAVIR_RITONAVIR

$DATA DATA.csv IGNORE=@

$SUBROUTINE ADVAN2 TRANS1

;-----------------------------------------------------------------------------------------------------

$PK

; ----------BETWEEN SUBJECT VARIABILITY(BSV)

BSVCL = ETA(1)

; ----------BETWEEN OCCASSION VARIABILITY(BOV)

BOVBIO = 0

BOVKA = 0

BOVLAG = 0

IF(OCCASSION.EQ.1) BOVBIO = ETA(2)

IF(OCCASSION.EQ.2) BOVBIO = ETA(3)

IF(OCCASSION.EQ.3) BOVBIO = ETA(4)

IF(OCCASSION.EQ.4) BOVBIO = ETA(5)

IF(OCCASSION.EQ.1) BOVKA = ETA(6)

IF(OCCASSION.EQ.2) BOVKA = ETA(7)

IF(OCCASSION.EQ.3) BOVKA = ETA(8)

IF(OCCASSION.EQ.4) BOVKA = ETA(9)

IF(OCCASSION.EQ.1) BOVLAG = ETA(10)

IF(OCCASSION.EQ.2) BOVLAG = ETA(11)

IF(OCCASSION.EQ.3) BOVLAG = ETA(12)

IF(OCCASSION.EQ.4) BOVLAG = ETA(13)

;--------- Allometric scaling and covariates ---------------------------------------------------------

; ------- Typical values of covariates

TVWT = 14 ;Weight

TVFFM = 10 ;Fat free mass

;--------- Allometric scaling

ALLMCL_WT = (WT/TVWT)**0.75

ALLMV_WT = (WT/TVWT)

ALLMCL_FFM = (FFM/TVFFM)**0.75

ALLMV_FFM = (FFM/TVFFM)

;------- Maturation of clearance

PGA = AGEM + 9 ; post gestational age, add 9 months

LOGPGA50 = THETA(1)

GAMMA = THETA(2)

MATCL = 0

IF (PGA>0) MATCL=1/(1+EXP(-EXP(GAMMA)*(LOG(PGA)-LOGPGA50)))

;--------------Method of drug administration on KA----------------------------------------------------

METHOD_KA = 1

IF (METHOD_OF_DRUG_ADMIN.EQ.1) METHOD_KA=THETA(11) ; CRUSHED

;--------------STUDY effect on Bio--------------------------------------------------------------------

STUDY_BIO = 1

IF (STUDY.EQ.2) STUDY_BIO=THETA(12) ;DNDI

;--------------LOPINAVIR_RITONAVIR on CL--------------------------------------------------------------

LPV_CL = 1

IF (LOPINAVIR_RITONAVIR.EQ.1) LPV_CL=THETA(13) ;On Lopinavir/ritonavir

;--------------Scaling BOV_Bio------------------------------------------------------------------------

BOVBIO_DN = BOVBIO

IF(SITE.EQ.6) BOVBIO_DN = BOVBIO*THETA(14) ;DNDI tygerberg site

;--------------Yesterday's Bio------------------------------------------------------------------------

YEST_BIO = 1

IF (OCCASSION.LE.1.AND.STUDY.EQ.3) YEST_BIO=THETA(15) ;Dosing occassions before PK day for DATIC

-----Typical values-----------------------------------------------------------------------------------

TVCL = THETA(3)*ALLMCL_FFM*MATCL*LPV_CL

TVV = THETA(4)*ALLMV_FFM

TVKA = THETA(5)*METHOD_KA

TVBIO = THETA(6)*YEST_BIO*STUDY_BIO

TVLAG = THETA(9)

;-----------Define parameters-------------------------------------------------------------------------

CL = TVCL*EXP(BSVCL) ; CLEARANCE

V = TVV; CENTRAL VOLUME

KA = TVKA*EXP(BOVKA) ; ABSORPTION RATE CONSTANT

BIO = TVBIO*EXP(BOVBIO) ; BIOAVAILABILITY

LAG =TVLAG*EXP(BOVLAG) ; LAG TIME

;-----------------------------------------------------------------------------------------------------

; re-parameterization

K = CL/V ;rate constant of elimination

ALAG1 = LAG

F1 = BIO

S2 = V

;-----------------------------------------------------------------------------------------------------

$ERROR

IPRED=A(2)/V

LLOQ = 0.200 ;Lower limit of quantification

CENS_THR = LLOQ

;India lab

IF (LAB.EQ.2) THEN

IPRED=(A(2)/V)*THETA(10)

LLOQ = 0.250

CENS_THR = 0.075 ;0.3*LLOQ inferred LOD = 30% of LLOQ

ENDIF

;DATIC

IF (STUDY.EQ.3) THEN

CENS_THR = 0.06 ;0.3*LLOQ inferred LOD = 30% of LLOQ

ENDIF

PROP = IPRED*THETA(7)

ADD = THETA(8)+(LLOQ*0.2)

; For CENS==1 (i.e. first CENSORED value in a series, which was imputed to CENS_THR/2), we add extra ;additive error on the concentrations, since the value in DV has been imputed and therefore more ;uncertain.

IF (ICALL/=4.AND.CENS==1) THEN

ADD = ADD +(CENS_THR*0.5)

ENDIF

NO_FIT = 0

; For CENS==2 (i.e. the trailing CENSORED values in a series that were imputed to CENS_THR/2), we ;don't want these to influence the fit,

; we only want them for simulation-based diagnostics.

IF (ICALL/=4.AND.CENS==2) THEN

PROP = 0

ADD = 10000000000

NO_FIT = 1

; Using this large error has the same effect as ignoring, expect the record is still there, so I can ;use it in VPCs

ENDIF

W = SQRT(ADD**2+PROP**2)

; Protective code

IF (W.LE.0.000001) W=0.000001

IRES=DV-IPRED

IWRES=IRES/W

Y = IPRED + W*ERR(1)

; To prevent simulation (ICALL==4) of negative values. It set a positive lower bound for Y, so that VPCs in the log-scale can be plotted

IF (ICALL==4.AND.Y<=CENS_THR) THEN

Y = CENS_THR/2

ENDIF

;-----------------------------------------------------------------------------------------------------

$THETA

2.42562 ; 1 PGA50 months [log]

1.23651 ; 2 GAMMA [log]

1.25347 ; 3 CL [L/h]

10.7794 ; 4 V [L]

1.89619 ; 5 KA [1/h]

1 FIX ; 6 BIO

0.0756404 ; 7 PROP []

0 FIX ; 8 ADD [mg/L]

0.154065 ; 9 LAG

1.34752 ; 10 Lab_India vs UCT

1.99281 ; 11 MODE_KA

0.722167 ; 12 DNDI_BIO

1.47945 ; 13 DN_LPV_CL

2.67136 ; 14 TYG_BOVBIO

0.641779 ; 15 DAT_YEST_BIO

;-----------------------------------------------------------------------------------------------------

$OMEGA BLOCK(1)

0.0491627 ; 1 BSV CL

;-----------------------------------------------------------------------------------------------------

$OMEGA BLOCK(1)

0.0911206 ; 2 BOVBIO

$OMEGA BLOCK(1) SAME

$OMEGA BLOCK(1) SAME

$OMEGA BLOCK(1) SAME

;-----------------------------------------------------------------------------------------------------

$OMEGA BLOCK(1)

0.510963 ; 7 BOVKA

$OMEGA BLOCK(1) SAME

$OMEGA BLOCK(1) SAME

$OMEGA BLOCK(1) SAME

;-----------------------------------------------------------------------------------------------------

$OMEGA BLOCK(1)

0.843296 ; 11 BOVLAG

$OMEGA BLOCK(1) SAME

$OMEGA BLOCK(1) SAME

$OMEGA BLOCK(1) SAME

;-----------------------------------------------------------------------------------------------------

$SIGMA 1 FIX

;-----------------------------------------------------------------------------------------------------

$ESTIMATION MSFO=run001.msf MAXEVAL=9999 PRINT=1 METHOD=1 INTER

NOABORT NSIG=3 NONINFETA=1 ETASTYPE=1
